# Targeting astrocytic Dp71 attenuates BBB disruption after traumatic brain injury through WTAP-associated m^6^A regulation of MMP2

**DOI:** 10.1126/sciadv.aed8653

**Published:** 2026-07-03

**Authors:** Jiheng Wang, Chenzhu Zhao, Bei Liu, Zhuoyang Wang, Yueru Hou, Yuankun Chen, Wenxing Cui, Kailu Li, Jinyuan Meng, Ge Ren, Tao Xin, Zihao Liu, Xun Wu, Yingxi Wu, Yafei Xue, Gang Zhu, Zhihong Li, Shunnan Ge, Dayun Feng, Yan Qu, Tianzhi Zhao

**Affiliations:** ^1^ Department of Neurosurgery, Tangdu Hospital, Fourth Military Medical University, Xi’an, 710038 Shaanxi, China.; ^2^ Medical School, Northwest University, Xi’an, 710068 Shaanxi, China.; ^3^ Laboratory of Gene Therapy, Department of Biochemistry, College of Life Sciences, Shaanxi Normal University, Xi’an, 710062 Shaanxi, China.; ^4^ Department of Neurosurgery, The First Affiliated Hospital of Shandong First Medical University, Jinan 250014, China.; ^5^ Department of Neurosurgery, Shandong Provincial Hospital, Shandong First Medical University, Jinan 250021, China.

## Abstract

Blood-brain barrier (BBB) disruption is a major pathological feature of traumatic brain injury (TBI) that contributes to secondary damage and poor neurological recovery. Although astrocytes are essential for BBB homeostasis, the molecular basis of astrocyte-associated BBB dysfunction after TBI remains unclear. Here, we found that astrocytic dystrophin protein 71 (Dp71) expression was reduced after TBI in both patients and mouse models. In mice, further experimental down-regulation of astrocytic Dp71 attenuated secondary BBB disruption and was accompanied by reduced astrocyte activation, inflammatory cell infiltration, and matrix metalloproteinase-2 (MMP2) release. Mechanistically, nuclear Dp71 interacted with Wilms tumor 1–associated protein (WTAP) and influenced its ubiquitination, leading to changes in the N^6^-methyladenosine (m^6^A) modification, RNA stability, and expression of *MMP2* messenger RNA. In addition, biomimetic nanovesicles coated with astrocyte membranes enabled targeted delivery of small interfering RNA targeting Dp71 (siDp71) to astrocytes and reduced MMP2 release and BBB damage after TBI, suggesting a potential therapeutic strategy for mitigating BBB injury after TBI.

## Teaser

Targeting astrocytic Dp71 attenuates secondary BBB disruption after TBI via WTAP-associated m^6^A regulation of MMP2.

## INTRODUCTION

Traumatic brain injury (TBI) is a major cause of mortality and long-term disability worldwide, yet effective interventions to improve neurological outcomes remain limited (*1*, *2*). Blood-brain barrier (BBB) disruption is a prominent pathological feature of TBI that contributes to poor recovery (*3*–*6*). Specifically, primary TBI injury refers to the immediate mechanical damage to brain tissue, blood vessels, and the BBB caused by trauma, whereas secondary TBI injury involves a delayed pathological cascade characterized by neuroinflammatory, vascular, and multicellular responses that contribute to long-term complications such as seizures and cognitive impairment (*4*). Secondary injury develops over hours to days after trauma and is characterized by progressive BBB breakdown, which together with these pathological responses exacerbates edema, neuronal loss, and long-term neurological complications (*5*–*7*).

Astrocytes are major cellular components of the central nervous system and essential regulators of BBB homeostasis (*8*–*14*). Beyond their roles in neural circuit function, lipid and ion homeostasis, and vascular regulation, astrocytes are structural and functional components of the neurovascular unit and influence BBB permeability (*11*–*14*). Recent single-cell transcriptomic studies have shown that astrocytes undergo marked gene expression changes after TBI (*15*), indicating that astrocyte-associated molecular programs may contribute to BBB dysfunction during injury progression.

The dystrophin gene (*DMD*) gives rise to multiple transcript variants (*16*, *17*). In the central nervous system, the dystrophin protein 71 (Dp71) isoform is abundantly expressed in astrocytes, whereas the full-length Dp427 isoform is not detected in these cells (*18*–*20*). Dp71 has been linked to brain development, cytoskeletal organization, and the regulation of astrocyte morphology and motility (*21*–*24*). Loss of Dp71 reduces astrocytic process complexity (*25*), supporting a role for Dp71 in maintaining astrocyte structural organization. However, whether astrocytic Dp71 influences BBB pathology after TBI and the molecular basis for such effects remains unclear.

Here, we found that astrocytic Dp71 was reduced after TBI in both human specimens and mouse models. In mice, further experimental targeting of astrocytic Dp71 attenuated secondary BBB disruption and was accompanied by reduced astrocyte activation, inflammatory cell infiltration, and matrix metalloproteinase-2 (MMP2) release. These effects were further linked to a Wilms tumor 1–associated protein (WTAP)–associated regulatory pathway involving N^6^-methyladenosine (m^6^A) modification of *MMP2* mRNA, and biomimetic nanovesicle–mediated delivery of small interfering RNA targeting Dp71 (siDp71) reduced BBB damage after TBI. Together, these findings support the involvement of astrocytic Dp71 in secondary BBB disruption after TBI and suggest its potential as a therapeutic target for secondary BBB damage after TBI.

## RESULTS

### Astrocytic Dp71 is reduced after TBI in human and mouse samples and is associated with BBB disruption

**Fig. 1.**
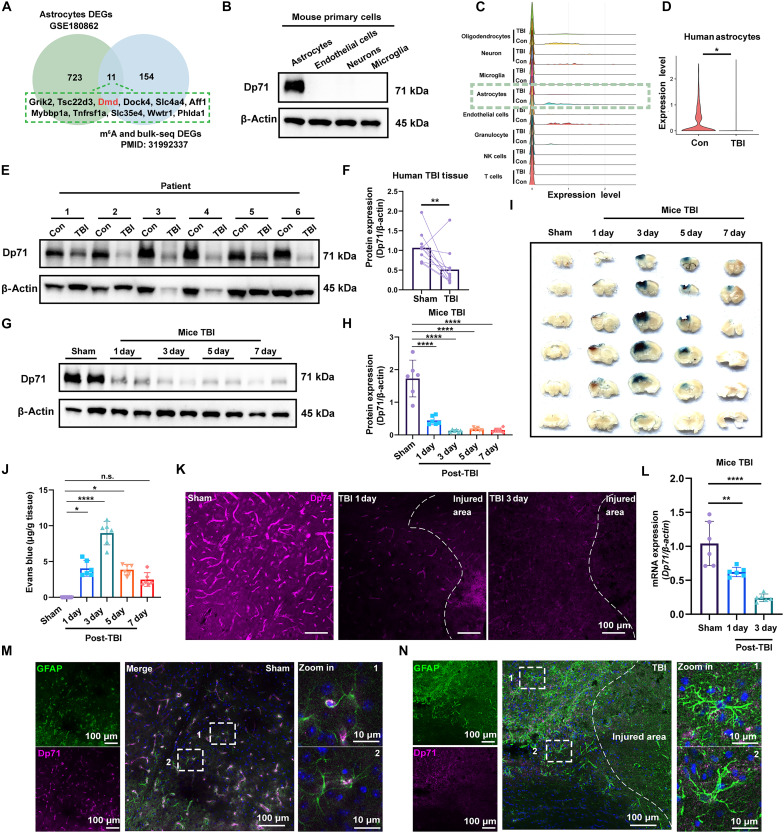
Dp71 expression is decreased in astrocytes of mice and patients after TBI. (**A**) Venn analysis of DEGs in astrocytes after TBI (downloaded from GSE180862) and DEGs from m^6^A sequencing and transcriptome sequencing of the injured cerebral cortex in TBI mice (PMID: 31992337). (**B**) Western blot analysis of Dp71 protein expression in primary astrocytes, primary endothelial cells, primary neurons, and primary microglia. (**C** and **D**) Single-cell RNA sequencing analysis of gene expression profiles in astrocytes from patients with TBI and controls (Con). (**E** and **F**) Western blot analysis of Dp71 protein expression levels in patients (*n* = 11 per group; Mann-Whitney *U* test). (**G** and **H**) Western blot analysis of Dp71 protein expression levels in mice at different time points after TBI [*n* = 6 per group; one-way analysis of variance (ANOVA)]. (**I** and **J**) Evans blue staining analysis of BBB permeability in mice at different time points after TBI (*n* = 6 per group; Kruskal-Wallis test with Dunn’s multiple-comparison test). (**K**) Immunofluorescence staining analysis of Dp71 expression in the TBI-injured brain region at different time points after TBI (*n* = 6 per group). Scale bars, 100 μm. (**L**) Real-time polymerase chain reaction (PCR) analysis of Dp71 mRNA expression levels at different time points after TBI (*n* = 6 per group; one-way ANOVA). (**M** and **N**) Immunofluorescence staining analysis of glial fibrillary acidic protein (GFAP) and Dp71 colabeling in the sham and TBI groups (*n* = 6). Scale bars, 100 and 10 μm (zoom). Results are expressed as means ± SD. *P < 0.05, **P < 0.01, ****P < 0.0001, and n.s., not significant.

Astrocytes undergo marked transcriptional changes during the acute and subacute phases after TBI (*15*). To prioritize candidates for further study, we integrated astrocyte transcriptomic changes after TBI with genes showing differential m^6^A modification (*26*), a major posttranscriptional RNA modification—the most prevalent internal modification of eukaryotic RNAs, dynamically regulated by “writers,” “erasers,” and “readers” to modulate RNA metabolism (*27*).This analysis yielded 11 overlapping differentially expressed genes (DEGs; Fig. 1A). Among these candidates, we focused on Dp71, the major brain isoform of *DMD*, because it is abundantly expressed in the brain and was predominantly detected in astrocytes compared with endothelial cells, neurons, and microglia (Fig. 1B). In our previous single-cell RNA sequencing data from patients with TBI (*28*), *DMD* expression was reduced in astrocytes (Fig. 1, C and D). Because these data did not distinguish the *Dp71* isoform, we further examined Dp71 expression in cortical tissues from 11 patients with TBI and corresponding control cortical tissues from patients without TBI who underwent brain surgery for glioma or cavernous hemangioma. Dp71 protein expression was markedly reduced in the injured region (Fig. 1, E and F).

In the mouse TBI model, Dp71 expression showed a similar pattern and progressively decreased during the acute phase, reaching its lowest level at day 3 and partially recovering thereafter (Fig. 1, G and H). Evans blue extraction showed that changes in Dp71 protein levels were temporally associated with Evans blue concentration (Fig. 1, I and J), with peak Evans blue extravasation and the lowest Dp71 level both observed at day 3 after TBI. This time point was therefore used for subsequent analyses of the relationship between Dp71 and BBB integrity after TBI.

Immunofluorescence showed reduced DMD immunoreactivity around the injured region after TBI (Fig. 1K). Dp71 could not be distinguished from Dp427 by immunofluorescence because of antibody limitations (*29*). We further examined *Dp71* mRNA expression and found a pattern consistent with the protein data (Fig. 1L). Colabeling of DMD with aquaporin 4 (AQP4), ionized calcium-binding adapter molecule 1 (Iba1), and neuronal nuclei (NeuN) showed no detectable signal in microglia or neurons (fig. S1A), whereas Dp71 immunoreactivity colocalized with AQP4, indicating enrichment in astrocytic endfeet. Costaining with glial fibrillary acidic protein (GFAP) further showed lower Dp71 expression in activated astrocytes after TBI (Fig. 1, M and N).

### Astrocyte-specific Dp71 knockdown attenuates secondary BBB disruption and cerebral edema after TBI

**Fig. 2.**
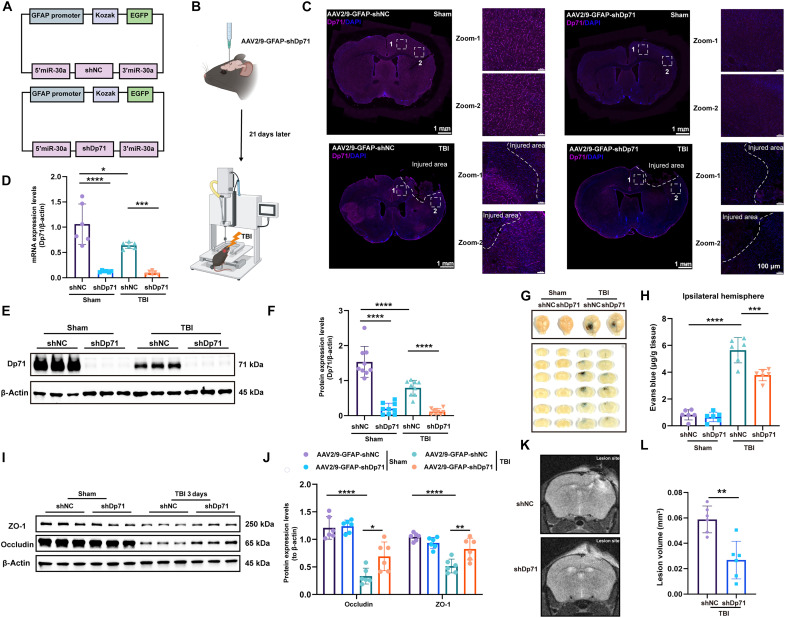
Knockdown of Dp71 in astrocytes alleviates secondary damage to the BBB after TBI. (**A**) Schematic of the AAV encoding astrocyte-specific Dp71-shRNA. EGFP, enhanced green fluorescent protein; shNC, short hairpin RNA negative control; 5ʹ miR-30a, 5ʹ microRNA-30a. (**B**) Schematic of the procedure for TBI induction 21 days after AAV injection. Created in BioRender. Jiheng, W. (2026); https://BioRender.com/by3e1z6. (**C**) Immunofluorescence staining analysis of Dp71 expression in mouse brain (*n* = 6 per group). (**D**) Real-time PCR analysis of *Dp71* mRNA in the injured area of mouse brain 3 days after TBI (*n* = 6 per group; two-way ANOVA). (**E** and **F**) Western blot analysis of Dp71 protein expression levels in the injured area of mouse brain (*n* = 9 per group; two-way ANOVA). (**G** and **H**) Evans blue staining analysis of BBB permeability in mice with astrocyte-specific Dp71 knockdown at 3 days after TBI (*n* = 6 per group; two-way ANOVA). (**I** and **J**) Western blot analysis of ZO-1 and Occludin (BBB permeability markers) protein expression levels 3 days after TBI (*n* = 6 per group; two-way ANOVA). (**K** and **L**) MRI analysis of brain edema and lesion volume (*n* = 6 per group; Student’s *t* test). Results are expressed as means ± SD. *P < 0.05, **P < 0.01, ***P < 0.001, ****P < 0.0001, and n.s., not significant.

Astrocyte-specific knockdown of Dp71 was achieved using a GFAP promoter–driven short hairpin RNA (shRNA) adeno-associated virus 2/9 (AAV2/9) (Fig. 2, A and B). Dp71 expression was reduced in mice injected with AAV2/9–GFAP promoter–short hairpin RNA targeting Dp71 (shDp71), as shown by immunofluorescence (Fig. 2C), quantitative polymerase chain reaction (qPCR; Fig. 2D), and Western blot (Fig. 2, E and F). In the mouse TBI model, astrocytic Dp71 knockdown attenuated BBB permeability, as indicated by reduced Evans blue extravasation in gross brain sections (Fig. 2G) and lower Evans blue content in the injured hemisphere (Fig. 2H). Consistent with this, the expression of the tight junction proteins Occludin and zonula occludens-1 (ZO-1), the core structural proteins of the BBB tight junctions (*30*), was increased in the Dp71 knockdown group (Fig. 2, I and J). Astrocytic Dp71 knockdown also attenuated edema-related changes after TBI. Magnetic resonance imaging (MRI) analysis showed reduced T2-weighted imaging -hyperintense lesion volume and less severe swelling–related changes (Fig. 2, K and L). Hematoxylin and eosin (H&E) and Nissl staining further showed milder tissue injury and more surviving neurons in the Dp71 knockdown group (fig. S2, A to C). Coimmunofluorescence staining for NeuN and terminal deoxynucleotidyl transferase–mediated deoxyuridine triphosphate nick end labeling further showed fewer apoptotic neurons after TBI (fig. S2, D and E), and Dp71 knockdown also attenuated cerebral edema after TBI (fig. S2F). Consistently, similar results were obtained with injection of cholesterol-conjugated small interfering RNA (siRNA; fig. S3, A and B), which was used to improve siRNA stability in vivo and facilitate cellular uptake.

Opposite effects were observed in mice with astrocyte-specific Dp71 overexpression following injection of AAV2/9–GFAP Promoter–Dp71 into the cerebral cortex 21 days before TBI. Elevated Dp71 expression in the overexpression group was verified by immunofluorescence (fig. S4A), qPCR (fig. S4B), and Western blot (fig. S4, C and D). Astrocytic Dp71 overexpression increased Evans blue extravasation (fig. S4, E and F). Meanwhile, Occludin and ZO-1 expression was significantly decreased in Dp71 overexpression group (fig. S4, G and H). MRI analysis revealed increased brain lesion volume and more severe swelling–related changes in the Dp71 overexpression group post-TBI (fig. S4, I and J). Dp71 overexpression also exacerbated cerebral edema (fig. S4K) and was associated with more severe tissue injury and fewer surviving neurons on H&E and Nissl staining (fig. S5, A to C).

### Astrocyte-specific Dp71 knockdown reduces astrocyte reactivity, immune cell infiltration, and neurological deficits after TBI

**Fig. 3.**
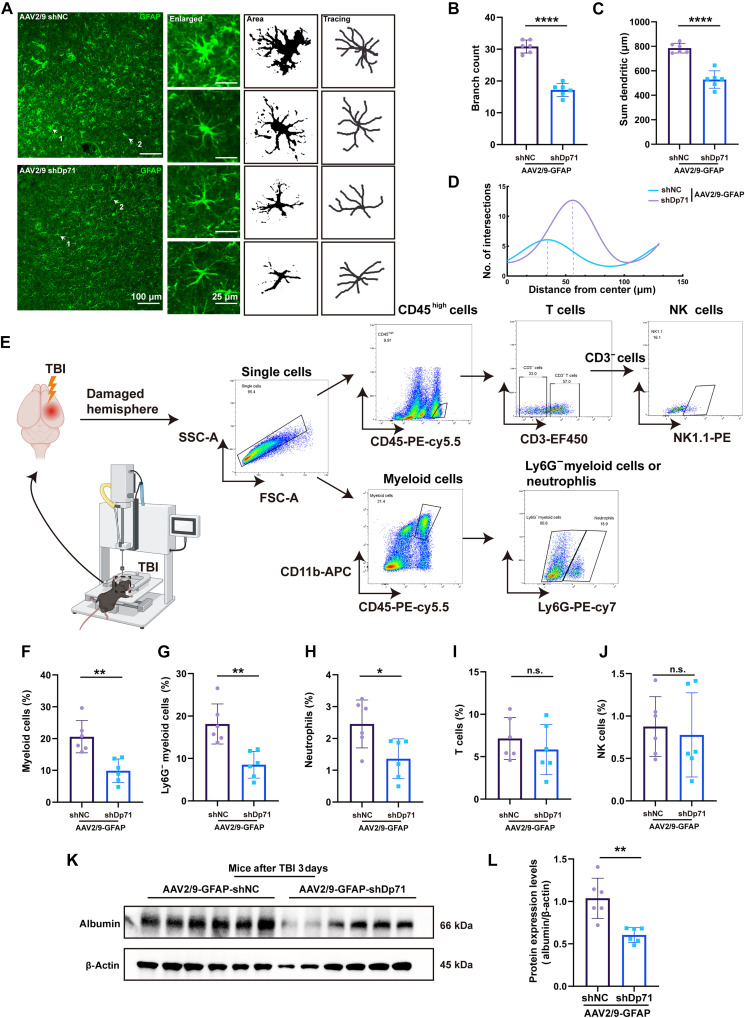
Dp71 astrocytic knockdown inhibits astrocyte reactivity and reduces immune cell infiltration after TBI. (**A**) Immunolabeling and morphological analysis of astrocytes in the injured area of mouse brain of shNC (short hairpin RNA negative control) and shDp71 mice at 3 days after TBI (*n* = 6 per group; Student’s *t* test; data are presented as the mean for each group). Scale bars, 100 and 25 μm (enlarged). (**B**) Dendritic branch count of the astrocytes. (**C**) The sum intersections of the astrocytes. (**D**) Variation trend of the intersections following distance from center. SSC-A, side scatter area; FSC-A, Forward scatter area. (**E**) Representative gating strategy for flow cytometric analysis of immune cells in the brain. Created in BioRender. Jiheng, W. (2026); https://BioRender.com/v3ntw94. (**F** to **J**) Quantification of myeloid cells, Ly6G^−^myeloid cells, neutrophils, T cells, and NK cells in the injured hemispheres of the brain after TBI (*n* = 6 per group; Student’s *t* test). (**K** and **L**) Western blot analysis of albumin expression levels at 3 days after TBI (*n* = 6 per group; Student’s *t* test). Results are expressed as means ± SD. *P < 0.05, **P < 0.01, ****P < 0.0001, and n.s., not significant.

Astrocyte-specific Dp71 knockdown reduced astrocyte reactivity after TBI. GFAP immunostaining in the frontal cortex showed reduced astrocytic branching and process complexity in the shDp71 group (Fig. 3A). Specifically, the number of branches (Fig. 3B) and total dendritic length (Fig. 3C) were decreased, and Sholl analysis further showed reduced dendritic complexity after Dp71 knockdown (Fig. 3D). Flow cytometry further showed reduced infiltration of immune cells in the injured hemisphere after TBI. Cell populations were identified using the following markers: CD45^+^ CD11b^+^ for myeloid cells, CD45^+^ CD11b^+^ Ly6G^+^ for neutrophils, CD45^+^ CD11b^+^ Ly6G^−^ (lymphocyte antigen 6 complex locus G negative) for Ly6G^−^ myeloid cells, CD45^high^ CD3^+^ for T cells, and CD45^high^ CD3^−^ NK1.1^+^ for natural killer (NK) cells (Fig. 3E).

In the shDp71 group, the numbers of myeloid cells (Fig 3F), Ly6G^−^ myeloid cells (Fig. 3G), and neutrophils (Fig. 3H) were lower after TBI, whereas T cell and NK cell numbers were not significantly changed (Fig. 3, I and K). Western blot analysis of albumin further supported reduced plasma protein extravasation and improved BBB integrity in Dp71-knockdown mice after TBI (Fig. 3, L and M).

Astrocyte-specific Dp71 knockdown was also associated with improved neurological performance after TBI. In the rotarod test, shDp71 mice showed longer fall latency (fig. S6A). In the adhesive tape removal test, shDp71 mice showed shorter times to touch and remove the tape (fig. S6, B and C). In the open field test, shDp71 mice traveled a greater total distance and showed higher average speed (fig. S6, D to F). Together, these behavioral results indicated faster neurological improvement after TBI in shDp71 mice.

### Dp71 regulates MMP2 mRNA and protein expression in astrocytes

**Fig. 4.**
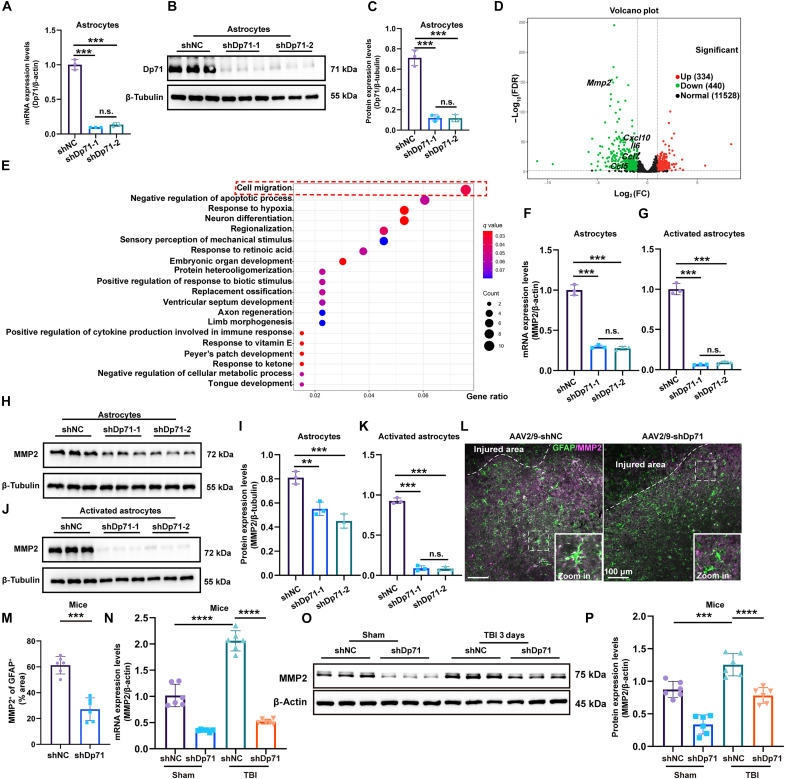
Dp71 regulates the RNA expression level of MMP2. (**A**) Real-time PCR analysis of Dp71 mRNA expression in astrocytes infected with shNC, shDp71-1, and shDp71-2 lentiviruses (*n* = 3 per group; one-way ANOVA). (**B** and **C**) Western blot analysis of Dp71 protein expression in astrocytes infected with shNC, shDp71-1, and shDp71-2 lentiviruses (*n* = 3 per group; one-way ANOVA). (**D**) Volcano plot showing decreased expression of typical inflammatory genes in the shDp71 group. FDR, false discovery rate. (**E**) Gene Ontology (GO) enrichment analysis of DEGs (molecular function module). (**F** and **G**) Real-time PCR analysis of *MMP2* mRNA expression in astrocytes and activated astrocytes (*n* = 3 per group; one-way ANOVA). (**H** to **K**) Western blot analysis of MMP2 protein expression in astrocytes and activated astrocytes (*n* = 3 per group; one-way ANOVA). (**L** and **M**) Immunofluorescence analysis of GFAP and MMP2 colabeling in the injured region of mice at 3 days after TBI (*n* = 6 per group; Student’s *t* test). Scale bars, 100 μm. (**N**) Real-time PCR analysis of *MMP2* mRNA expression in the injured region of TBI mice and the corresponding cerebral cortex of sham mice (*n* = 6 per group; two-way ANOVA). (**O** and **P**) Western blot analysis of MMP2 protein expression in the injured region of TBI mice and the corresponding cerebral cortex of sham mice (*n* = 6 per group; two-way ANOVA). Results are expressed as means ± SD. **P < 0.01, ***P < 0.001, ****P < 0.0001, and n.s., not significant.

To investigate the molecular mechanisms by which Dp71 regulates astrocyte activation and BBB-associated changes, we generated astrocytes stably expressing shNC (short hairpin RNA negative control), shDp71-1, and shDp71-2 via lentivirus transduction. shDp71-1 and shDp71-2 are two independent shRNAs designed to target distinct coding regions of the *Dp71* transcript. These independent shRNA constructs were used to minimize potential off-target effects of gene silencing. Efficient knockdown of Dp71 was confirmed at both the mRNA and protein levels (Fig. 4, A to C). In the in vitro Transwell coculture system, we examined the effect of Dp71 expression in astrocytes on tight junction proteins in bEnd.3 endothelial cells. Immunofluorescence showed that knockdown of Dp71 in astrocytes increased ZO-1 staining intensity in bEnd.3 cells (fig. S7, A and B). Western blot analysis further confirmed that Dp71 knockdown increased the protein levels of ZO-1 and Occludin in bEnd.3 cells (fig. S7, C and D). Conversely, overexpression of Dp71 in astrocytes achieved by lentiviral transduction (fig. S8, A and B) decreased the levels of ZO-1 and Occludin in bEnd.3 endothelial cells (fig. S8, C to F).

RNA sequencing analysis was then performed to identify DEGs (Fig. 4D), which revealed decreased expression of inflammatory factors such as *MMP2*, interleukin-6 (*IL-6*), and tumor necrosis factor–α (*TNF-α*). Gene Ontology (GO) enrichment analysis (molecular function module) demonstrated that cell migration–related pathways were enriched among DEGs (Fig. 4E), with *MMP2* showing a prominent expression change. We further focused on activated astrocytes, as astrocytes become robustly activated after TBI. Activated astrocytes are the primary mediators of inflammatory response and glial scar formation and play a key role in the progression of BBB disruption and secondary brain injury after TBI (*31*).While MMP2 expression was up-regulated at both the mRNA and protein levels in activated astrocytes (fig. S9, A to C), Dp71 knockdown consistently reduced MMP2 expression at both the mRNA and protein levels in both resting and activated astrocytes (Fig. 4, F to K). Immunofluorescence staining further demonstrated lower MMP2 expression within GFAP^+^ astrocytes in the brains of shDp71 mice (Fig. 4, L and M). Western blot and qPCR analyses confirmed that Dp71 knockdown significantly decreased *MMP2* mRNA (Fig. 4N) and protein (Fig. 4, O and P) expression levels in the mouse brain after TBI. Conversely, qPCR and Western blot analyses revealed higher MMP2 expression in Dp71-overexpressing mice following TBI (fig. S9, D to F).

Further studies showed that in shDp71 astrocytes and in TBI mice receiving cholesterol-conjugated Dp71 siRNA via injection, the protein levels of the inflammatory factors IL-6 and TNF-α were lower than those in the corresponding control cells and mice (fig. S9, G to J). Reverse transcription (RT)–qPCR further showed that the mRNA expression levels of chemokines including C-C motif chemokine ligand (*CCL5*), *CCL7*, and *CXCL10* were significantly decreased in shDp71 astrocytes and in mice (fig. S9, K and L). Furthermore, enzyme-linked immunosorbent assay (ELISA) results demonstrated that Dp71 knockdown reduced the expression of CCL5, CCL7, CXCL10, TNF-α, and IL-6 both in vitro and in vivo (fig. S9, M to V).

### Nuclear Dp71 interacts with WTAP in astrocytes

**Fig. 5.**
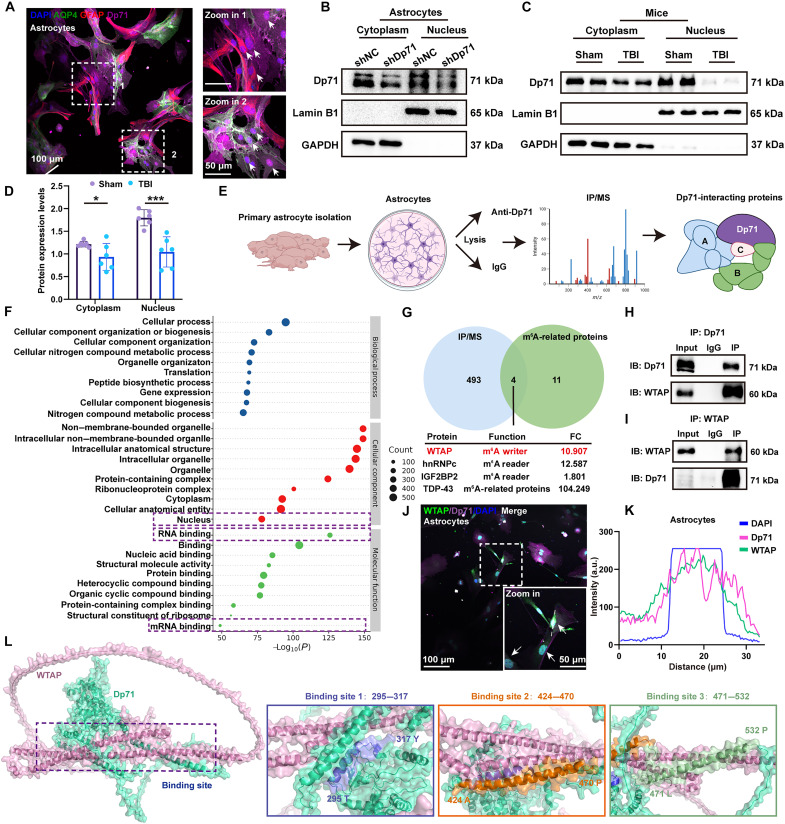
Dp71 interacts with WTAP in the nuclei of astrocytes. (**A**) Immunofluorescence analysis of GFAP, AQP4, Dp71, and 4′,6-diamidino-2-phenylindole (DAPI) colabeling, with DAPI serving as a nuclear marker. Scale bars, 100 and 50 μm (enlarged). (**B**) Representative Western blot analysis of Dp71 localization via nuclear-cytoplasmic fractionation in shNC and shDp71 groups (*n* = 3 per group). (**C** and **D**) Representative Western blot analysis of Dp71 localization via nuclear-cytoplasmic fractionation in mice at 3 days after TBI (*n* = 6 per group; Student’s *t* test). (**E**) Schematic diagram of Dp71-interacting proteins detected by IP/MS. Created in BioRender. Jiheng, W. (2026); https://BioRender.com/igeixur. *m/z*, mass/charge ratio. (**F**) GO enrichment analysis of Dp71-binding proteins detected by IP/MS. (**G**) Venn analysis of Dp71-binding proteins and m^6^A-related proteins. FC, fold change. (**H** and **I**) Immunoblot (IB) analysis of Co-IP for Dp71 and WTAP in astrocytes (*n* = 3). (**J** and **K**) Immunofluorescence analysis of Dp71 and WTAP colocalization in astrocytes (*n* = 3). Scale bars, 100 and 50 μm (enlarged). a.u., arbitrary units. (**L**) Molecular docking and binding site analysis of Dp71 and WTAP. Results are expressed as means ± SD. *P < 0.05, ***P < 0.001.

To investigate how Dp71 regulates MMP2 expression in astrocytes after TBI, we characterized the subcellular localization of Dp71. Immunofluorescence and nucleocytoplasmic fractionation showed that Dp71 was distributed in both the cytoplasm and nucleus of primary astrocytes (Fig. 5, A and B). In mouse brain tissue, Dp71 was also detected in both compartments, and nuclear Dp71 expression was reduced after TBI (Fig. 5, C and D). This nuclear localization suggested that Dp71 might participate in transcriptional or posttranscriptional regulation.

To identify molecular partners potentially involved in Dp71-mediated regulation of MMP2, we performed immunoprecipitation–mass spectrometry (IP/MS) analysis in primary astrocytes (Fig. 5E). GO enrichment analysis of the IP/MS results revealed that the major binding proteins of Dp71 were enriched for RNA binding activity and nuclear localization, suggesting a possible association with RNA regulatory processes (Fig. 5F). Among the Dp71-binding proteins, Venn diagram analysis identified four m^6^A-related proteins—WTAP, heterogeneous nuclear ribonucleoprotein C (hnRNPc), insulin-like growth factor 2 mRNA-binding protein 2 (IGF2BP2), and TAR DNA-binding protein 43 (TDP-43) (Fig. 5G). Endogenous reciprocal coimmunoprecipitation (Co-IP) experiments validated the interactions between Dp71 and hnRNPc, IGF2BP2, or TDP-43 (fig. S10). Among these proteins, WTAP, as a component of the m^6^A methyltransferase complex (m^6^A writer), is directly involved in m^6^A modification (*32*, *33*). Therefore, the interaction between Dp71 and WTAP was the focus of subsequent investigations. Co-IP experiments confirmed the interaction between Dp71 and WTAP in astrocytes (Fig. 5, H and I). In addition, in primary astrocytes, immunofluorescence assays also demonstrated that Dp71 and WTAP colocalized in the nucleus (Fig. 5, J and K). To further characterize the Dp71-WTAP interaction, we used AlphaFold3 to predict the three-dimensional structures of Dp71 and WTAP, followed by molecular docking analysis. Protein Data Bank in Europe Protein Interfaces, Surfaces and Assemblies (PDBePISA) analysis indicated that residues 295 to 317, 424 to 470, and 471 to 532 of Dp71 may represent key regions for WTAP binding. The intermolecular forces governing the binding of these three regions are mediated primarily by hydrogen bonds, and the detailed information of these three key binding sites is presented in Fig. 5L.

### Dp71 influences WTAP protein stability and global m^6^A levels in astrocytes

**Fig. 6.**
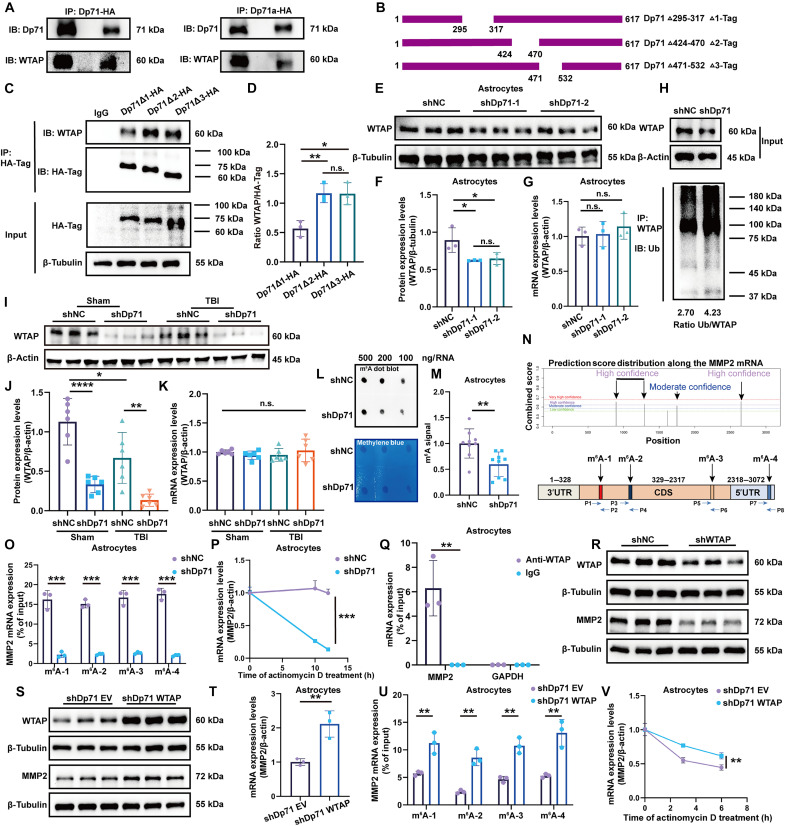
Dp71 regulates WTAP’s ubiquitination in astrocytes, which, in turn, regulates the m^6^A level of MMP2 to modulate its expression. (**A**) Co-IP for exogenous Dp71-HA, Dp71a-HA, and WTAP in astrocytes (*n* = 3). (**B**) Schematic of truncated Dp71 mutants. (**C** and **D**) Co-IP for interactions between truncated Dp71 mutants and WTAP (*n* = 3; one-way ANOVA). (**E** and **F**) Western blot analysis of WTAP protein expression levels in astrocytes (*n* = 3; one-way ANOVA). (**G**) Real-time PCR analysis of WTAP mRNA expression levels in astrocytes (*n* = 3; one-way ANOVA). (**H**) WTAP ubiquitination (Ub) assay in astrocytes treated with 20 μM MG-132 for 8 hours (*n* = 3). (**I** and **J**) Western blot analysis of WTAP protein in the injured mouse brain cortex at 3 days post-TBI (*n* = 6; two-way ANOVA). (**K**) Real-time PCR analysis of WTAP mRNA in the injured mouse brain cortex at 3 days post-TBI (*n* = 6; two-way ANOVA). (**L** and **M**) m^6^A dot blot analysis for m^6^A levels (*n* = 9; Student’s *t* test). (**N**) Prediction of m^6^A modification sites in *MMP2* mRNA using the SRAMP tool. 3′UTR, 3′ untranslated region; CDS, coding sequence. (**O**) Methylated RNA immunoprecipitation (MeRIP) analysis of m^6^A modification levels on *MMP2* mRNA (*n* = 3; Student’s *t* test). (**P**) Real-time PCR analysis of MMP2 mRNA stability (*n* = 3; Student’s *t* test). h, hours. (**Q**) RIP-qPCR analysis of the binding between WTAP protein and *MMP2* mRNA (*n* = 3; Student’s *t* test). (**R** and **S**) Western blot analysis of MMP2 protein expression levels (*n* = 3; Student’s *t* test). (**T**) Real-time PCR analysis of *MMP2* mRNA expression levels (*n* = 3; Student’s *t* test). (**U**) MeRIP analysis of *MMP2* mRNA m^6^A modification levels (*n* = 3; Student’s *t* test). (**V**) Real-time PCR analysis of *MMP2* mRNA stability (*n* = 3; Student’s *t* test). Results are expressed as means ± SD. *P < 0.05, **P < 0.01, ***P < 0.001, ****P < 0.0001, and n.s., not significant.

Different Dp71 transcripts were separately transfected into astrocytes. Co-IP results showed that exogenously expressed Dp71–hemagglutinin (HA) and Dp71a-HA both interacted with WTAP (Fig. 6A). On the basis of molecular docking predictions, Dp71 mutants with deletions of key binding sites were designed to identify the specific binding region mediating the Dp71-WTAP interaction (Fig. 6B): The Dp71 mutants lacking amino acids 295 to 317, 424 to 470, and 471 to 532 were named Dp71Δ1-HA, Dp71Δ2-HA, and Dp71Δ3-HA, respectively. Co-IP results demonstrated that the interaction between Dp71Δ1-HA and WTAP was significantly attenuated (Fig. 6, C and D), suggesting that the amino acid sequence spanning positions 295 to 317 of Dp71 may represent a key region for binding to WTAP.

Furthermore, Western blotting analysis revealed that the WTAP protein level was significantly down-regulated in shDp71 astrocytes (Fig. 6, E and F), while its mRNA level remained unchanged (Fig. 6G). IP experiments further showed that WTAP ubiquitination was increased in shDp71 astrocytes (Fig. 6H). Cycloheximide (CHX) assays revealed that WTAP declined more rapidly in shDp71 astrocytes than in control cells, suggesting that Dp71 knockdown decreases WTAP protein stability (fig. S11, A and B). In vivo, WTAP protein was also significantly down-regulated in the brain tissues of shDp71 mice after TBI (Fig. 6, I and J), whereas no significant difference was detected in its mRNA level (Fig. 6K), consistent with the in vitro results. m^6^A dot blot analysis further showed that global m^6^A levels were reduced in shDp71 astrocytes (Fig. 6, L and M).

### Dp71 regulates MMP2 mRNA stability through WTAP-dependent m^6^A modification

We next examined whether Dp71 regulates the m^6^A modification and expression of MMP2 mRNA through WTAP. Single-cell RNA sequencing showed that although *DMD* (the gene encoding Dp71) expression was reduced in astrocytes after TBI, *DMD*-high astrocyte subpopulations remained detectable (fig. S11C), and these cells exhibited higher *MMP2* expression than *DMD*-low astrocytes (fig. S11D). Analysis of the Gene Expression Profiling Interactive Analysis (GEPIA) database further showed positive correlations of *DMD* and *WTAP* expression with *MMP2* expression in the cerebral cortex and hippocampus (fig. S11, E and F). SRAMP (*34*) predicted three high-confidence and one moderate-confidence m^6^A sites on *MMP2* mRNA (Fig. 6N), and RNA secondary structure analysis identified the locations of these sites (fig. S11G). Methylated RNA immunoprecipitation (MeRIP)–qPCR showed that methylation levels at all four predicted m^6^A sites on *MMP2* mRNA were reduced in shDp71 astrocytes (Fig. 6O), and actinomycin D assays further showed reduced *MMP2* mRNA stability (Fig. 6P). Consistent with these findings, *MMP2* mRNA and protein expression were decreased in shDp71 astrocytes.

To further test whether Dp71-dependent regulation of MMP2 requires WTAP, RIP-qPCR confirmed binding between *WTAP* and *MMP2* mRNA (Fig. 6Q). In WTAP-overexpression astrocytes, both the protein and mRNA levels of *MMP2* were increased (fig. S11, H and I). In WTAP-knockdown astrocytes, MMP2 protein expression was decreased (Fig. 6R), MMP2 mRNA was reduced (fig. S11J), *MMP2* mRNA stability was impaired (fig. S11K), and m^6^A modification of *MMP2* mRNA was reduced (fig. S11L). In WTAP-rescued shDp71 astrocytes, the protein level (Fig. 6S), mRNA expression (Fig. 6T), and m^6^A modification level (Fig. 6U) of MMP2 were restored, and *MMP2* mRNA stability was partially recovered (Fig. 6V). These results indicate that Dp71 regulates *MMP2* expression through WTAP-dependent effects on m^6^A modification and mRNA stability.

The IGF2BP family functions as m^6^A readers that regulate the stability of target RNAs (*35*). To identify the m^6^A reader for *MMP2* mRNA, we generated astrocytes with knockdown of IGF2BP1, IGF2BP2, or IGF2BP3 using two distinct shRNAs for each factor. qPCR and Western blot analyses confirmed the knockdown efficiency (fig. S12, A to I). Only IGF2BP3 knockdown reduced MMP2 expression at both the mRNA and protein levels (fig. S13, A to I), whereas knockdown of IGF2BP1 or IGF2BP2 had no significant effect. RIP-qPCR confirmed binding between IGF2BP3 and *MMP2* mRNA (fig. S14A), and actinomycin D assays further showed reduced *MMP2* mRNA stability after IGF2BP3 knockdown (fig. S14B). IGF2BP3 knockdown did not alter Dp71 mRNA or protein expression (fig. S14, C and D), supporting a role for IGF2BP3 in maintaining *MMP2* mRNA stability in astrocytes.

To assess the functional relevance of MMP2 downstream of Dp71, we treated mice with astrocyte-specific Dp71 overexpression with an MMP2 inhibitor after TBI. MMP2 inhibition attenuated the increase in Evans blue extravasation induced by astrocytic Dp71 overexpression (fig. S15, A and B). Consistently, the down-regulation of Occludin and ZO-1 was also alleviated by MMP2 inhibition (fig. S15, C and D).

### Biomimetic astrocyte membrane–fused nanovesicles delivering siDp71 attenuate BBB disruption and cerebral edema after TBI

**Fig. 7.**
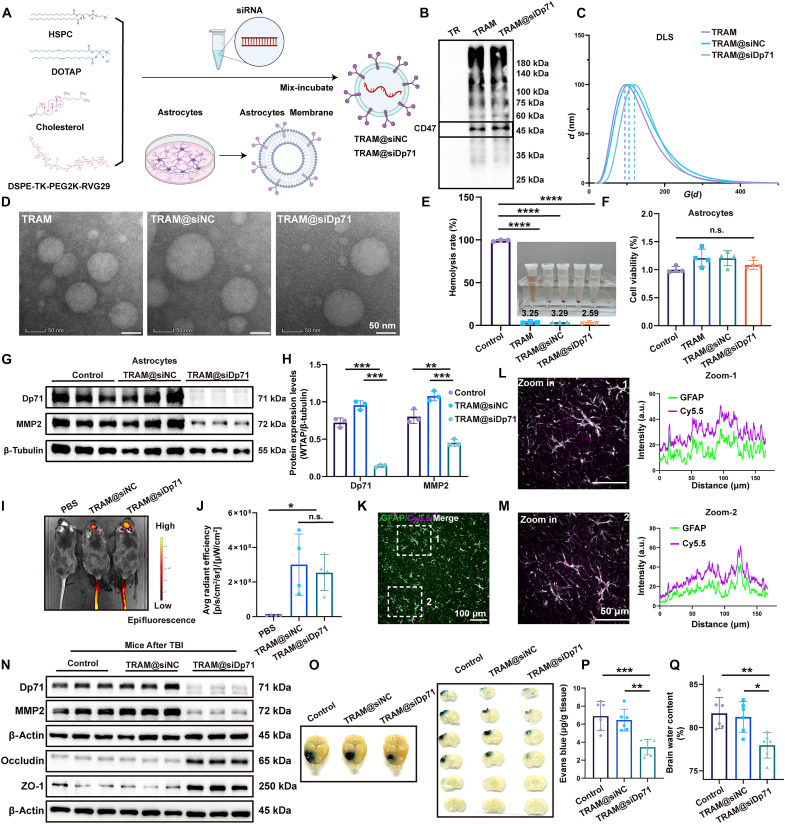
Biomimetic astrocyte cell membrane–fused nanovesicle-mediated delivery of siDp71 protects the BBB and alleviates cerebral edema. (**A**) Schematic diagram of TRAM@siNC and TRAM@siDp71 synthesis. TRAM was prepared by fusing liposomes with astrocyte membrane and conjugating with the RVG29 peptide. Created in BioRender. Jiheng, W. (2026); https://BioRender.com/p9xmg9o. (**B**) Western blot analysis of the protein expression of CD47 (a cell membrane–specific molecule) on the surface of TRAM@siNC and TRAM@siDp71 (*n* = 3 per group). (**C**) Particle size analysis of TRAM@siNC and TRAM@siDp71 (*n* = 3 per group). DLS, dynamic light scattering. (**D**) Transmission electron microscopy (TEM) observation of TRAM@siNC and TRAM@siDp71. Scale bars, 50 nm (*n* = 3 per group). (**E**) Hemolytic activity assay of TRAM, TRAM@siNC, and TRAM@siDp71 (*n* = 3 per group; one-way ANOVA). (**F**) Cell Counting Kit-8 (CCK-8) assay for the cytotoxicity of TRAM, TRAM@siNC, and TRAM@siDp71 (*n* = 4 per group; one-way ANOVA). (**G** and **H**) Western blot analysis of Dp71 and MMP2 protein expression in astrocytes in vitro after uptake of TRAM@siNC and TRAM@siDp71 (*n* = 3 per group; one-way ANOVA). (**I** and **J**) Real-time fluorescence imaging to detect the distribution of Cy5.5 in mouse brain regions after intravenous injection of TRAM@siNC and TRAM@siDp71 (*n* = 4 per group; one-way ANOVA). Avg, average. (**K** to **M**) Immunofluorescence analysis of the colocalization of GFAP and Cy5.5. Scale bars, 100 and 50 μm (zoom) (*n* = 6 per group). (**N**) Western blot analysis of Dp71, MMP2, Occludin, and ZO-1 protein expression in mice after TBI (*n* = 6 per group; one-way ANOVA). (**O** and **P**) Evans blue staining analysis of BBB permeability in mice after TBI (*n* = 6 per group; one-way ANOVA). (**Q**) Assessment of cerebral edema in mice at 3 days after TBI by quantifying brain water content (*n* = 6 per group; one-way ANOVA). Results are expressed as means ± SD. *P < 0.05, **P < 0.01, ***P < 0.001, ****P < 0.0001, and n.s., not significant.

Although lentiviral and adeno-associated viral approaches showed efficacy in mouse experiments, their translational feasibility remains limited (*36*). To extend our mechanistic findings toward a therapeutic strategy, we developed an astrocyte-targeted nanovesicle for siDp71 delivery, termed TRAM@siDp71. This system was designed to combine BBB penetration with astrocyte targeting through RVG29 modification and astrocyte membrane fusion (Fig. 7A). Western blot analysis confirmed that astrocyte membrane protein was successfully anchored on the surface of the nanoparticles (Fig. 7B), and characterization showed uniform particle size distribution and regular morphology (Fig. 7, C and D). Drug loading and encapsulation efficiencies of siDp71 and Cy5.5 were evaluated (fig. S16, A and B). To assess the safety of TRAM@siDp71, we intravenously injected mice with TRAM@siDp71, TRAM@siNC, or vehicle. No significant differences were detected in hematological indices or serum biochemical parameters among groups (fig. S16, C to Q), and no significant toxicity was observed in major organs (fig. S17). In vitro, neither TRAM@siNC nor TRAM@siDp71 caused hemolysis (Fig. 7E) or significant cytotoxicity in astrocytes (Fig. 7F), HT22 neuronal cells, bEnd.3 endothelial cells, or BV2 microglia (fig. S18, A to C).

In vitro functional assays showed that TRAM@siDp71 effectively mediated siRNA-dependent gene silencing and significantly down-regulated the expression of Dp71 and MMP2 at both the mRNA (fig. S19A) and protein levels (Fig. 7, G and H). Following intravenous injection, in vivo fluorescence imaging showed clear Cy5.5 signals in the brain at 24 hours, indicating BBB penetration by both TRAM@siNC and TRAM@siDp71 (Fig. 7, I and J). Immunofluorescence staining results further showed that Cy5.5 signals exhibited significant colocalization with GFAP (Fig. 7K); colocalization analysis results confirmed that both TRAM@siNC and TRAM@siDp71 showed efficient targeting to astrocytes (Fig. 7, L and M).

We next examined the effects of TRAM@siDp71 after TBI. RT-qPCR showed that TRAM@siDp71 reduced *Dp71* and *MMP2* mRNA expression in the injured brain region (fig. S19B), and Western blot analysis showed reduced Dp71 and MMP2 protein expression together with increased Occludin and ZO-1 levels (Fig. 7N). Evans blue staining showed reduced extravasation in the TRAM@siDp71 group (Fig. 7, O and P), consistent with attenuation of secondary BBB disruption, and cerebral edema was also reduced after TBI (Fig. 7Q). In behavioral testing, TRAM@siDp71-treated mice showed longer fall latency in the rotarod test (fig. S19C), shorter times to touch and remove the tape in the adhesive tape removal test (fig. S19, D and E), and greater total travel distance and higher average speed in the open field test (fig. S19, F to H). These results support the in vivo safety, astrocyte-targeting capability, and therapeutic potential of TRAM@siDp71 after TBI.

## DISCUSSION

After TBI, BBB disruption contributes to secondary injury (*4*, *6*). Astrocytes are integral components of the neurovascular unit whose roles in BBB pathophysiology remain incompletely understood (*37*–*40*). In this study, we found that astrocytic Dp71 was reduced after TBI in both human TBI specimens and mouse models. In mice, further experimental down-regulation of astrocytic Dp71 attenuated secondary BBB disruption and was linked to WTAP-associated m^6^A regulation of MMP2.

Dp71 has been linked to cell proliferation, differentiation, metabolism, and inflammatory responses. In the central nervous system, Dp71-deficient astrocytes show enhanced proliferation and metabolic capacity together with an attenuated inflammatory response to lipopolysaccharide/interferon-γ stimulation (*41*). Similarly, astrocytic Dp71 knockdown in vivo reduced astrocyte reactivity after TBI, reflected by decreased branch number, total branch length, and process complexity (Fig. 3, A to D).Previous work in the Middle Cerebral Artery Occlusion/Reperfusion (MCAO/R) model showed that Dp71 is down-regulated because of increased ubiquitination and that restoration of Dp71 reduces cerebral edema (*42*). In contrast, our data indicate that after TBI, astrocytic Dp71 down-regulation occurs at the transcriptional rather than protein level (Fig. 1, D and L), which further down-regulation attenuates edema-related changes (Fig. 2, K and L, and fig. S2F), and that astrocytic overexpression of Dp71 aggravates BBB damage and related injury-associated changes after TBI (figs. S4 and S5). This difference may reflect the distinct temporal and pathological features of the TBI and MCAO/R models. Because our analyses focused on 3 days after TBI, when Dp71 expression was lowest and BBB damage was most severe, the role of Dp71 during later phases of BBB repair remains unclear.

Astrocytes show marked transcriptional and functional diversity after injury, and our single-cell RNA sequencing data revealed divergent *DMD* expression across astrocyte clusters in the acute phase after TBI, with DMD-high subpopulations coupled to higher *MMP2* expression. These observations suggest that the regulation and effects of Dp71 may not be uniform across astrocyte subpopulations. Further studies will be needed to define the cell subtype–specific regulation of Dp71 after TBI.

Previous studies showed that cordycepin reduces MMP2/MMP9 expression and attenuates BBB damage after TBI (*43*), and astrocyte membrane–fused nanovesicles have been used for astrocyte-directed delivery (*44*). Using a similar biomimetic system, we delivered siDp71 to astrocytes and observed reduced MMP2 expression and BBB damage after TBI, supporting the feasibility of astrocyte-directed Dp71 targeting after TBI.

In summary, our study shows that astrocytic Dp71 is reduced after TBI in both human specimens and mouse models, whereas further experimental targeting of astrocytic Dp71 in mice attenuates secondary BBB disruption, along with reductions in astrocyte activation, inflammatory cell infiltration, and MMP2 release. These findings indicate that endogenous posttraumatic Dp71 reduction and experimental Dp71 targeting are not functionally equivalent but instead reflect a context-dependent role of astrocytic Dp71 in secondary BBB injury. Mechanistically, the protective effect of Dp71 targeting was associated with a WTAP-dependent pathway involving m^6^A modification of *MMP2* mRNA. Moreover, biomimetic nanovesicle–mediated delivery of siDp71 reduced BBB damage after TBI, further supporting the therapeutic potential of this strategy. Together, our findings provide previously unidentified insight into astrocyte-mediated regulation of BBB integrity after TBI and suggest that Dp71-targeted modulation may represent a potential therapeutic approach for secondary BBB damage.

## MATERIALS AND METHODS

### Datasets

Single-cell resolution datasets (GSE180862) (*15*) were used to analyze system spatiotemporal dynamics of TBI and identify DEGs of astrocytes in the cortical region during the acute phase of TBI after 24 hours. Bulk RNA sequencing (Bulk-seq) datasets were obtained from the published article (PMID: 31992337) (*26*).Venn diagram is used to identify overlapping genes.

### Mouse TBI model and patients with TBI

C57Bl/6 mice (8 to 10 weeks old, 25 to 30 g) were obtained from the Laboratory Animal Center of the Fourth Military Medical University. The induction of a TBI in mice began with anesthetizing via inhalation of a mixture of 5% isoflurane (RWD Life Science) and 100% oxygen at a flow rate of 1 liter/min. To maintain anesthesia, the isoflurane concentration was reduced to 2% (Sigma-Aldrich) and continuously administered.

After anesthesia, mice were fixed in stereotaxic frame. A surgical incision was made along the midline to expose the skull, and a precise 4.0-mm-diameter craniotomy window was created on the right parietal lobe. Subsequently, 2-mm-diameter flat-tipped impactor was used to vertically impact the exposed cortex, with the impactor set at a velocity of 3.0 m/s, an impact depth of 1.5 mm, and a dwell time of 0.85 s. The success of the controlled cortical impact (CCI) model was confirmed by the presence of visible cortical contusion. Following the successful establishment of the CCI model, the skin incision was sutured. Mice in the sham group underwent only craniotomy without CCI induction (*45*).

All experiments involving human participants were approved by the Ethics Committee of Tangdu Hospital, Fourth Military Medical University (ethics approval no. GKJ-Y-202503-077). Written informed consent was obtained from individual(s) included in this study. Experimental protocols were approved by the Ethics Committee of the Fourth Military Medical University and complied with the guidelines of the National Institutes of Health (NIH) Guide for the Care and Use of Laboratory Animals (institutional number: 202103-006; IACUC-20210920).

### Immunofluorescence

After dissection, the brain was carefully removed and immersed in 4% paraformaldehyde for 10 hours to ensure adequate fixation. Following fixation, the brain underwent dehydration processes in 15 and 30% sucrose solutions, respectively. The dehydrated brain tissues were then sectioned into 25-μm-thick slices, which were permeabilized with 0.1% Triton X-100 for 30 min and blocked with 5% donkey serum for 2 hours to reduce nonspecific antibody binding. Subsequently, the sections were incubated with primary antibodies: chicken anti-GFAP (PA1-10004, Thermo Fisher Scientific; diluted 1:500), rabbit anti-DMD (12715-1-AP, Proteintech, diluted 1:200), mouse anti-AQP4 (74806, CST; diluted 1:100), mouse anti-Iba1 (EPR16589, Abcam; diluted 1:200), and rabbit anti-MMP2 (A11144, ABclonal; 1:100). After primary antibody incubation, the brain sections were then treated with corresponding secondary antibodies to facilitate visualization under a microscope. 4′,6-diamidino-2-phenylindole (DAPI) staining was used to stain nuclei.

Cells were fixed with 4% paraformaldehyde for 15 min at room temperature, permeabilized with 0.1% Triton X-100 for 10 min, and blocked with 5% donkey serum for 1 hour. Then cells were incubated overnight at 4°C with primary antibodies: chicken anti-GFAP (PA1-10004, Thermo Fisher Scientific; diluted 1:500), rabbit anti–ZO-1 (21773-1-AP, Proteintech; diluted 1:200), rabbit anti-DMD (12715-1-AP, Proteintech; diluted 1:200), rabbit anti-WTAP (10200-1-AP, Proteintech; diluted 1:400), and mouse anti-AQP4 (74806, CST; diluted 1:100). After washing with phosphate-buffered saline (PBS), cells were incubated with corresponding fluorescence-conjugated secondary antibodies for 1 hour at room temperature in the dark. Nuclei were counterstained with DAPI.

Images were obtained using a laser confocal microscope.

### BBB permeability assays and brain water content assessment

A 2.0% Evans blue solution (4 ml/kg; Sigma-Aldrich) was administered to mice via tail vein injection. Two hours postinjection, the ipsilateral half-brain containing the damaged region was dissected, and its weight was recorded. The harvested tissue was homogenized in *N*,*N*-dimethylformamide, followed by centrifugation to collect the supernatant. The concentration of Evans blue in the supernatant was quantified by measuring the absorbance at 620 nm (*46*).

Small-animal MRI scans were performed using a 7.0-T scanner. Lesion volumes were quantified by manually outlining hyperintense areas on each T2-weighted slice and calculating the sum of these areas multiplied by the slice thickness. Two independent investigators blinded to group assignments analyzed the data.

After the brain was removed from the animal, its wet weight was immediately measured with precision. Subsequently, the harvested brain was placed in an oven and dried at a constant temperature of 110°C for 48 hours to ensure complete evaporation of tissue moisture, followed by reweighing to obtain its dry weight. The brain water content was calculated using the formula: brain water content (%) = [(wet weight − dry weight)/wet weight] × 100%.

### Methylated RNA immunoprecipitation–qPCR

MeRIP experiments were performed using BersinBio m^6^A MeRIP Kit (BersinBio, China), according to the manufacturer’s protocol. Briefly, 100 μg of RNA is extracted from astrocytes and then fragmented, after which the RNA is collected. The samples were incubated overnight with anti-m^6^A and immunoglobulin G (IgG) antibodies. qRT-PCR is used to detect the level of *MMP2* mRNA m^6^A methylation. The primers used were as follows:

*MMP2* m^6^A 1 forward: TGGGAGCATGGAGATGGATAC

*MMP2* m^6^A 1 reverse: GTGAGAATCTCCCCCAACACC

*MMP2* m^6^A 2 forward: TGGCACCACCGAGGACTAT

*MMP2* m^6^A 2 reverse: CCACAGTGGACATAGCGGTC

*MMP2* m^6^A 3 forward: CACACCAACACTGGGACCTG

*MMP2* m^6^A 3 reverse: TCACCACGGATCTGAGCGAT

*MMP2* m^6^A 4 forward: TGACCTTTTTATGGCTTTCAGCA

*MMP2* m^6^A 4 reverse: CCCCATGCCAGGCTGTTA

### RNA immunoprecipitation–qPCR

RIP experiments were performed using the Magna RIP RNA-Binding Protein Immunoprecipitation Kit (Millipore). Anti-WTAP (10200-1-AP, Proteintech) and anti-IGF2BP3 (14642-1-AP, Proteintech) antibodies were used for IP. *MMP2* mRNA levels were detected by qRT-PCR.

### Flow cytometric analysis of immune cell infiltration

Mice were humanely euthanized, and ipsilateral hemispheres containing the damaged region were immediately harvested into ice-cold Dulbecco’s modified Eagle’s medium (DMEM). The tissues were enzymatically digested with collagenase D (0.66 mg/ml; COLLD-RO, Roche) and deoxyribonuclease I (8 U/ml; EN0521, Thermo Fisher Scientific) for 30 min at 37°C to dissociate into single-cell suspensions. Immune cells were isolated using the Percoll density gradient method and resuspended in Cell Staining Buffer (E-CK-A107, Elabscience). The following fluorochrome-conjugated antibodies were used for flow cytometry: PerCP/Cyanine5.5 Anti-Mouse CD45 Antibody (E-AB-F1136J, Elabscience); APC Anti-Mouse/Human CD11b Antibody (E-AB-F1081E, Elabscience); PE/Cyanine7 Anti-Mouse Ly6G Antibody (E-AB-F1108H, Elabscience); PE Anti-Mouse CD161/NK1.1 Antibody (E-AB-F0987D, Elabscience); and Elab Fluor Violet 450 Anti-Mouse CD3 Antibody (E-AB-F1013UQ, Elabscience). After a 1-hour incubation with antibodies at 4°C in the dark, samples were analyzed using a flow cytometer, and data were processed with the FlowJo software.

### Assessments of sensorimotor and cognitive functions

#### Rotarod test

The rotarod test was used to evaluate motor balance and limb coordination in mice. Testing time points were set at baseline (pre-TBI) and on days 3, 5, 7, and 14 post-TBI. Mice were placed on a rotating rod, and the device accelerated steadily from 5 to 40 rpm over a 5-min period. Within the 14 days following TBI, each mouse was tested three to four times per day, with 5-min intervals between trials. The recorded metric was the mean latency to fall, calculated as the average daily duration the mice remained on the rod across the three to four trials at each pre- and postoperative time point.

#### Adhesive tape removal test

The adhesive tape removal test was used to assess sensory-motor function of the forepaws. Before testing, mice were acclimated for 60 s in a transparent Plexiglas cylinder (30 cm in height, 20 cm in diameter). A piece of tape was then applied to the hairless area of the left forepaw. The time taken for the mouse to first contact the tape (time to touch) and the time required to completely remove it (time to remove) were recorded. During the 14 days post-TBI, each mouse underwent three trials per day, and the average values were calculated. If a mouse failed to contact or remove the tape, then both times were recorded as the maximum cutoff value of 120 s.

#### Open field test

The open field test was conducted to evaluate general locomotor activity and exploratory behavior. Two days before formal testing, mice were acclimated to the open field arena for 5 min each day. On day 7 post-TBI, each mouse was individually placed in the central area of the arena, and its movement was recorded over a 10-min session. The floor of the arena was divided into four equal quadrants, and the distance traveled and velocity were analyzed for different zones. Laboratory lighting, temperature, and humidity were kept constant throughout testing. The apparatus was thoroughly cleaned with 75% ethanol or odorless detergent after each trial to eliminate residual odors.

### Cell culture and treatment

Primary astrocytes were isolated from the brains of neonatal C57BL/6J mice. Cells were maintained in a DMEM/F-12 medium (58-021-PBT, Corning) supplemented with 10% fetal bovine serum (A5256701,Thermo Fisher Scientific) under a humidified atmosphere of 5% CO_2_ at 37°C. TNF (30 ng/ml), IL-1α (3 ng/ml), and complement component 1q (C1q; 400 ng/ml) were added to primary astrocytes to induce their activation. Lentiviruses for knockdown of Dp71, WTAP, IGF2BP1, IGF2BP2, and IGF2BP3 were designated as shDp71, shWTAP, shIGF2BP1, shIGF2BP2, and shIGF2BP3, respectively, while the control lentivirus was named shNC. Lentiviruses for overexpression of Dp71 and WTAP were termed as Dp71 and WTAP, respectively, and the negative control lentivirus was termed as empty vector (EV). All lentiviruses were purchased from Tsingke (Tsingke Biotechnology Co. Ltd.), and the shRNA sequences are as follows:

shDp71-1:

GCATCATTTCTCTGTGTAACTCGAGTTACACAGAGAAATGATGCTTTTT

shDp71-2:

GGAGCTAGAGAGAATCCTACTCGAGTAGGATTCTCTCTAGCTCCTTTTTT

shWTAP:

GGAAAGTACACAGATCTTAATCTCGAGATTAAGATCTGTGTACTTTCCTTTTTT

shIGF2BP1-1:

GAAACACCTGACTCCAAAGTTCTCGAGAACTTTGGAGTCAGGTGTTTCTTTTTT

shIGF2BP1-2:

CGACCAAGTCATTGTTAAGATCTCGAGATCTTAACAATGACTTGGTCGTTTTTT

shIGF2BP2-1:

CTGTACCCTCATCACCATTTCCTCGAGGAAATGGTGATGAGGGTACAGTTTTTT

shIGF2BP2-2:

GCCGCATGATTCTTGAGATTACTCGAGTAATCTCAAGAATCATGCGGCTTTTTT

shIGF2BP3-1:

GCAGAGGATTCGTAAACTTCACTCGAGTGAAGTTTACGAATCCTCTGCTTTTTT

shIGF2BP3-2:

GCAGGGCCAACACATCAAACACTCGAGTGTTTGATGTGTTGGCCCTGCTTTTTT

The transwell coculture system was established using the bEnd.3 endothelial cell line and activated primary astrocytes. The bEnd.3 endothelial cell line was obtained from Fenghui Biotechnology Co. Ltd. Primary astrocytes were activated with TNF (30 ng/ml), IL-1α (3 ng/ml), and C1q before coculture (400 ng/ml). For the coculture model, bEnd.3 endothelial cells were seeded onto coverslips placed in a 24-well plate, while the activated astrocytes were added to the upper chamber of the Transwell system. After coculturing for different time points, the bEnd.3 endothelial cells were collected for subsequent immunofluorescence assays or Western blot analysis.

### AAV construction and stereotactic injection

All AAVs used in this study were purchased from Tsingke Biotechnology Co. Ltd. (1× 10^13^ vector genomes/ml). The constructs included AAV2/9–GFAP Promoter–shNC, AAV2/9–GFAP Promoter–shDp71 (for Dp71 knockdown, with the shRNA sequence identical to shRNA sequence 1 used in lentiviral vectors), AAV2/9–GFAP Promoter–EV, and AAV2/9–GFAP Promoter–Dp71 (for Dp71 overexpression). These AAVs were stereotaxically injected into the right parietal cortex of mice. Before injection, mice were anesthetized with isoflurane. The stereotaxic coordinates for AAV delivery were as follows

1.0 mm AP,+0.8 mm ML,–1.0 mm DV (1)

1.0 mm AP,+2.2 mm ML,–1.0 mm DV (2)

Here, AP (anterior-posterior) indicates the position relative to bregma along the rostrocaudal axis; ML (mediolateral) indicates the distance from the midline; and DV (dorsoventral) indicates the depth from the dura mater. A volume of 1 μl of AAV was delivered at each injection site at an infusion rate of ~0.2 μl/min. After each injection, the needle was left in place for 10 min to prevent backflow. In all experimental groups, TBI surgery was performed 21 days following AAV injection.

### Nuclear and cytoplasmic protein extraction

The Nuclear and Cytoplasmic Protein Extraction Kit (P0028, Beyotime) was used for cytoplasmic and nuclear protein extraction. According to the manufacturer’s instructions, the obtained cytoplasmic and nuclear proteins were quantified by Western blot analysis.

### m^6^A dot blot assay

Total RNA was extracted using the FastPure Cell/Tissue Total RNA Isolation Kit (RC101-01, Vazyme). The RNA samples were subjected to TGrade Lite (Tiangen, China) at 95°C for 3 min for denaturation. After that, the RNA samples were cross-linked onto a membrane through ultraviolet irradiation for 1 hour. Subsequently, the membrane was thoroughly washed with 1× SSC buffer and blocked with 5% nonfat dry milk. Overnight incubation at 4°C was then performed with a rabbit anti-m^6^A antibody (56593, CST; diluted 1:2000) to specifically detect m^6^A modifications on the RNA. Last, the membrane was incubated with a horseradish peroxidase–conjugated goat anti-rabbit IgG secondary antibody (AS014, ABclonal; diluted 1:10000). Methylene blue staining was used as a quality control step to confirm uniform RNA loading on the membrane.

### Coimmunoprecipitation

The cells were lysed with a suitable amount of cell lysis buffer intended for Western blotting and IP (P0013, Beyotime), supplemented with protease inhibitor cocktail, and incubated at 4°C for 2 hours. Following this, the supernatant lysates were harvested and subsequently incubated with primary antibodies overnight at 4°C. These antibodies included anti-Dp71 (12715-1-AP, Proteintech; diluted 1:100), anti-WTAP (10200-1-AP, Proteintech; diluted 1:100), anti-HNRNPC (11760-1-AP, Proteintech; diluted 1:100), anti-TDP-43 (0782-2-AP, Proteintech; diluted 1:100), and anti–HA-Tag (AE0086, Abclonal; diluted 1:100). After a 4-hour incubation at 4°C, the protein A/G magnetic beads (MedChemExpress, HY-K0202) were washed four times with the lysis buffer. Last, the proteins were eluted in SDS–polyacrylamide gel electrophoresis (PAGE) loading buffer and analyzed by immunoblotting.

### Quantitative reverse transcription polymerase chain reaction

Total RNA was extracted using the FastPure Cell/Tissue Total RNA Isolation Kit (RC101-01, Vazyme). cDNA was synthesized by RT with HiScript IV RT SuperMix (R423-01, Vazyme). qPCR was performed using the Taq Pro Universal SYBR qPCR Master Mix kit (Q712-02, Vazyme) according to the manufacturer’s instructions. *Dp71*, *MMP2*, *WTAP*, *IGF2BP1*, *IGF2BP2*, and *IGF2BP3* were all normalized to β-*actin*. The primers used were as follows:

*Dp71* forward: TCACTGCCTGTGAAACCCTT

*Dp71* reverse: TGTCATTTTGGGGTGGTCCC

*MMP2* forward: ACCTGAACACTTTCTATGGCTG

*MMP2* reverse: CTTCCGCATGGTCTCGATG

*WTAP* forward: ATGGCACGGGATGAGTTAATTC

*WTAP* reverse: TTCCCTTAAACCAGTCACATCG

*IGF2BP1* forward: ATGAAGGCCATCGAAACTTTCTC

*IGF2BP1* reverse: CATCGGAGCTGAGGTGGAATATT

*IGF2BP2* forward: TTCGACTGGACTGTCTGTGC

*IGF2BP2* reverse: TCGAGCGAGCTGTTTGATGT

*IGF2BP3* forward: ACGGAGACTGTGCATCTGTTTAT

*IGF2BP3* reverse: AATTCTTCCCTGAGCCTTGAACT

β-*actin* forward: GTGACGTTGACATCCGTAAAGA

β-*actin* reverse: GCCGGACTCATCGTACTCC

### Western blot analysis

The cell samples and brain tissues underwent lysis with RIPA Lysis Buffer (P0013B, Beyotime) to extract total protein. Subsequently, the protein concentration was determined using the bicinchoninic acid assay (BCA assay), and 20 μg of protein was separated by SDS-PAGE. Specifically, 12% polyacrylamide gels were used for the separation of target proteins with a molecular weight below 30 kDa, and 8% polyacrylamide gels were used for target proteins with a molecular weight above 30 kDa. The separated proteins were then transferred onto polyvinylidene fluoride membranes (Millipore, Shanghai, China). To minimize nonspecific binding, the membranes were blocked with 5% nonfat dried milk for 1 hour. Following blocking, the membranes were incubated overnight at 4°C with primary antibodies including anti-Dp71 (12715-1-AP, Proteintech; 1:10,000), anti-MMP2 (A19080, Abclonal; diluted 1:2000); anti–ZO-1 (21773-1-AP, Proteintech; diluted 1:10,000); Occludin (27260-1-AP, Proteintech; diluted 1:10,000), anti-WTAP (10200-1-AP, Proteintech; diluted 1:5000), anti-IGF2BP1 (22803-1-AP, Proteintech; diluted 1:5000), anti-IGF2BP2 (11601-1-AP, Proteintech; diluted 1:2000), anti-IGF2BP3 (14642-1-AP, Proteintech; diluted 1:5000), anti-ubiquitin (10201-2-AP, Proteintech; diluted 1:2000), anti-HNRNPC (11760-1-AP, Proteintech; diluted 1:5000), anti-TDP-43 (0782-2-AP, Proteintech; diluted 1:6000), anti-CD47 (A1838, Abclonal; diluted 1:1000); anti-Lamin B1 (12987-1-AP, Proteintech; diluted 1:10000), anti–glyceraldehyde-3-phosphate dehydrogenase (GAPDH; 60004-1-Ig, Proteintech; diluted 1:20,000), anti–β-tubulin (A12289, Abclonal; diluted 1:10,000), anti–β-actin (AC026, Abclonal; diluted 1:10000), anti–IL-1β (A22257, Abclonal; 1:1000), anti–TNF-α (A20851, Abclonal; 1:1000), anti–IL-6 (A0286, Abclonal; 1:1000), and anti–HA-Tag (AE0086, Abclonal; diluted 1:2000). After that, the membranes were washed thoroughly with tris-buffered saline with Tween 20. Subsequently, the membranes were incubated with the corresponding secondary antibodies for 1 hour. The secondary antibodies were detected using BeyoECL Plus (P0018S, Beyotime). Last, the chemiluminescent images were scanned and analyzed using the ImageJ NIH software, enabling the quantification and interpretation of the protein expression levels.

### Enzyme-linked immunosorbent assay

The levels of mouse IL-6 (YX-E20012-96, Sinobestbio), TNF-α (YX-E20533-96, Sinobestbio), CCL5 (YX-E20198-96, Sinobestbio), CCL7 (YX-E23527-96, Sinobestbio), and CXCL10 (YX-E20499-96, Sinobestbio) in tissue homogenates and cell lysates were measured using commercial ELISA kits according to the manufacturer’s instructions. The optical density was measured at 450 nm.

### RNA stability assay

To detect the stability of *MMP2* mRNA, actinomycin D (5 μg/ml; Sigma-Aldrich, USA) was added into cell culture medium. At diverse time points, cells were collected, and total RNA was isolated for qRT-PCR analysis.

### Protein stability assay

To evaluate the stability of WTAP protein, CHX (50 μg/ml; MCE, USA) was added to the cell culture medium. At the indicated time points, cells were collected, and total protein was extracted for Western blot analysis.

### Nissl and H&E staining

Nissl staining and H&E staining were performed to evaluate histopathological alterations and neuronal survival in brain tissues. Briefly, the harvested brain tissues were fixed in 4% paraformaldehyde, embedded in paraffin, and cut into 5-μm-thick sections. For H&E staining, the paraffin sections were deparaffinized in xylene, rehydrated through a graded series of ethanol, and stained with hematoxylin for 5 min and eosin for 30 s, followed by dehydration in graded ethanol, clearing in xylene, and mounting with neutral balsam. For Nissl staining, after deparaffinization and rehydration, the sections were stained with 0.1% cresyl violet acetate solution at 37°C for 15 min, differentiated with 95% ethanol for 30 s, dehydrated, cleared, and mounted as described above.

### MMP2 inhibitor treatment in vivo

The MMP2 inhibitor (HY-146754, MCE) was dissolved in 1% dimethyl sulfoxide in PBS. Mice were injected with the MMP2 inhibitor at a dose of 30 mg/kg at 2 hours after TBI to block MMP2 activity.

### Molecular docking

Amino acid sequences of mouse Dp71 (A2A9Z2) and WTAP (Q9ER69) were obtained from UniProt (www.uniprot.org/) (*47*). AlphaFold3 (https://alphafoldserver.com/welcome) (*48*) was used to build three-dimensional models of Dp71 and WTAP and to perform molecular docking between proteins. Discovery Studio was used to test the conformational rationality of the constructed molecular docking model. After obtaining the best quality model, PDBePISA (www.ebi.ac.uk/pdbe/pisa/) (*49*) was used to visualize the specific amino acid sites at the interaction interface of WTAP-Dp71 complex.

### Preparation of TRAM@siNC and TRAM@siDp71

The RVG29-modified cationic liposomes and cell membrane hybrid membrane@siRNA (siNC and siDp71) with Cy5.5 were prepared as follows: First, HSPC, DOTAP, DSPE-TK-PEG2K-RVG29, cholesterol, and Cy5.5 were codissolved in 3 ml of absolute ethanol and transferred into a round-bottom flask. Subsequently, siRNA was dissolved in citrate buffer (50 mM, pH 4.0) containing 25% ethanol, which was then slowly added to the aforementioned lipid solution. After thorough mixing, the mixture was incubated for 20 min, followed by sonication and extrusion through a liposome extruder with a 100-nm polycarbonate membrane. The astrocyte membrane (AM) was extracted using a Membrane and Cytosol Protein Extraction Kit (PC202, Epizyme) according to the manufacturer’s instructions. Then, 3 mg of the extracted AM was fused with the liposomes at 1:1 ratio (w/w). The resulting hybrid vesicles were dialyzed using a nanodialysis device equipped with a 10-nm pore size polycarbonate membrane. Last, a lyoprotectant was added, and the mixture was subjected to freeze drying.

### Characterization of TRAM@siNC and TRAM@siDp71

The particle size distribution of TRAM@siNC and TRAM@siDp71 was determined using NanoBrook 90Plus PALS (Brookhaven, USA). Morphological characteristics were observed using a TF20 transmission electron microscope (TEM; FEI, USA). The drug loading efficiency and encapsulation efficiency were calculated via spectrophotometry. The in vivo distribution and release profiles of the nanoparticles in mice were monitored using an IVIS Spectrum imaging system (Revvity, USA).

### Statistical analyses

All statistical analyses were performed using GraphPad Prism 9. Data are presented as the mean ± SD. Normality of data distribution was tested via the Shapiro-Wilk method. For normally distributed variables, an unpaired two-tailed Student’s *t* test was used to compare differences between two independent groups. For comparisons across multiple groups, one-way analysis of variance (ANOVA) followed by Bonferroni post hoc test was applied for experiments with a single independent variable, while two-way ANOVA was used for experiments with two independent variables to assess the main effects of each factor and their interaction, followed by Bonferroni post hoc test for prespecified pairwise comparisons. Nonnormally distributed variables were analyzed using the Kruskal-Wallis test with Dunn’s post hoc test (Bonferroni correction). All subjects were randomly assigned to experimental groups. The sample size for each experiment is indicated in the respective figure legends, and *P* < 0.05 was considered statistically significant.
