# Supplementary material for: Targeting astrocytic Dp71 attenuates BBB disruption after traumatic brain injury through WTAP-associated m6A regulation of MMP2
Source: Sci Adv. 2026 Jul 3;12(27):eaed8653. doi: 10.1126/sciadv.aed8653 (PMC13330862; doi:10.1126/sciadv.aed8653)
Supplement: Supplementary file 1 — Figs. S1 to S19 [file sciadv.aed8653_sm.pdf]

Science Advances
AAAS

Supplementary Materials for

Targeting astrocytic Dp71 attenuates BBB disruption after traumatic brain injury through WTAP-associated m^{6}A regulation of MMP2

Jiheng Wang *et al.*

Corresponding author: Dayun Feng, dayunfmmu@163.com; Yan Qu, yanqu0123@fmmu.edu.cn; Tianzhi Zhao, zhaotianzhi1981@163.com

Sci. Adv. **12**, eaed8653 (2026)
DOI: 10.1126/sciadv.aed8653

This PDF file includes:

Figs. S1 to S19

A

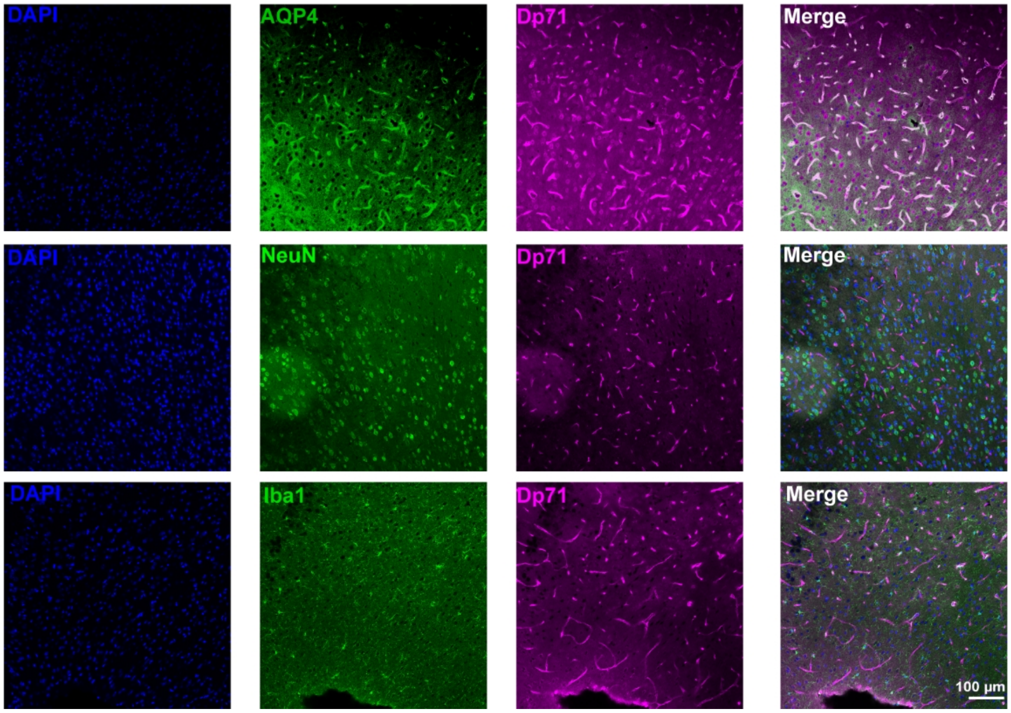

Fig. S1. Dp71 colocalizes with astrocytes.

A. Immunofluorescence analysis of the colocalization of Dp71 with the astrocyte marker AQP4, neuron marker NeuN, and microglia marker Iba1. Scale bars, 100 μm (n = 6 per group).

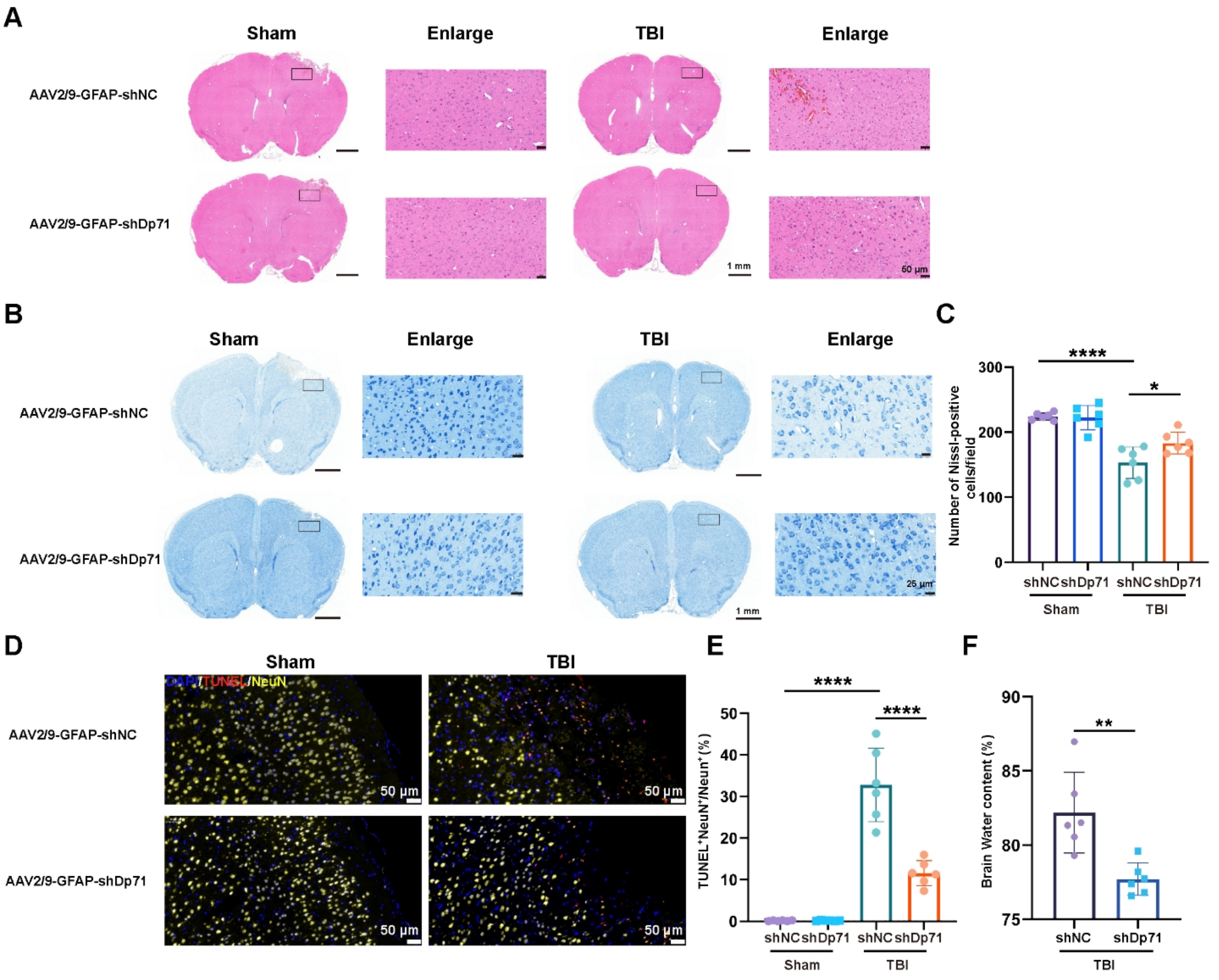

Fig. S2. Astrocyte-specific Dp71 knockdown mitigates TBI-induced brain injury, neuronal loss, neuronal apoptosis, and cerebral edema in mice.

A. HE staining in the perilesional cortex of Sham and TBI mice. Scale bars, 1 mm for low-magnification images, 50 μm for enlarged images.

B, C. Nissl staining in the perilesional cortex. Scale bars, 1 mm for low-magnification images, 25 μm for enlarged images (n = 6 per group, two-way ANOVA).

D, E. Immunofluorescence staining of TUNEL and NeuN in the perilesional cortex (n = 6 per group, two-way ANOVA).

F. Assessment of brain edema in mice 3 days after TBI by quantifying brain water content (n = 6 per group, Student's t-test).

Results are expressed as means ± SD. *P < 0.05, **P < 0.01, ***P < 0.001, ****P < 0.0001, NS, not significant.

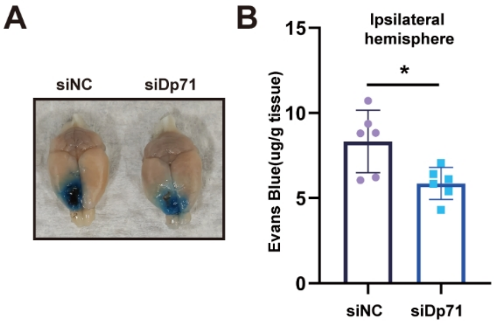

Fig. S3. Administration of cholesterol-conjugated siDp71 following TBI attenuates the secondary disruption of the BBB.

A, B. Evans blue staining analysis of BBB permeability in mice after TBI (n = 6 per group, Student's t-test).

Results are expressed as means ± SD. *P < 0.05, **P < 0.01, ***P < 0.001, ****P < 0.0001, NS, not significant.

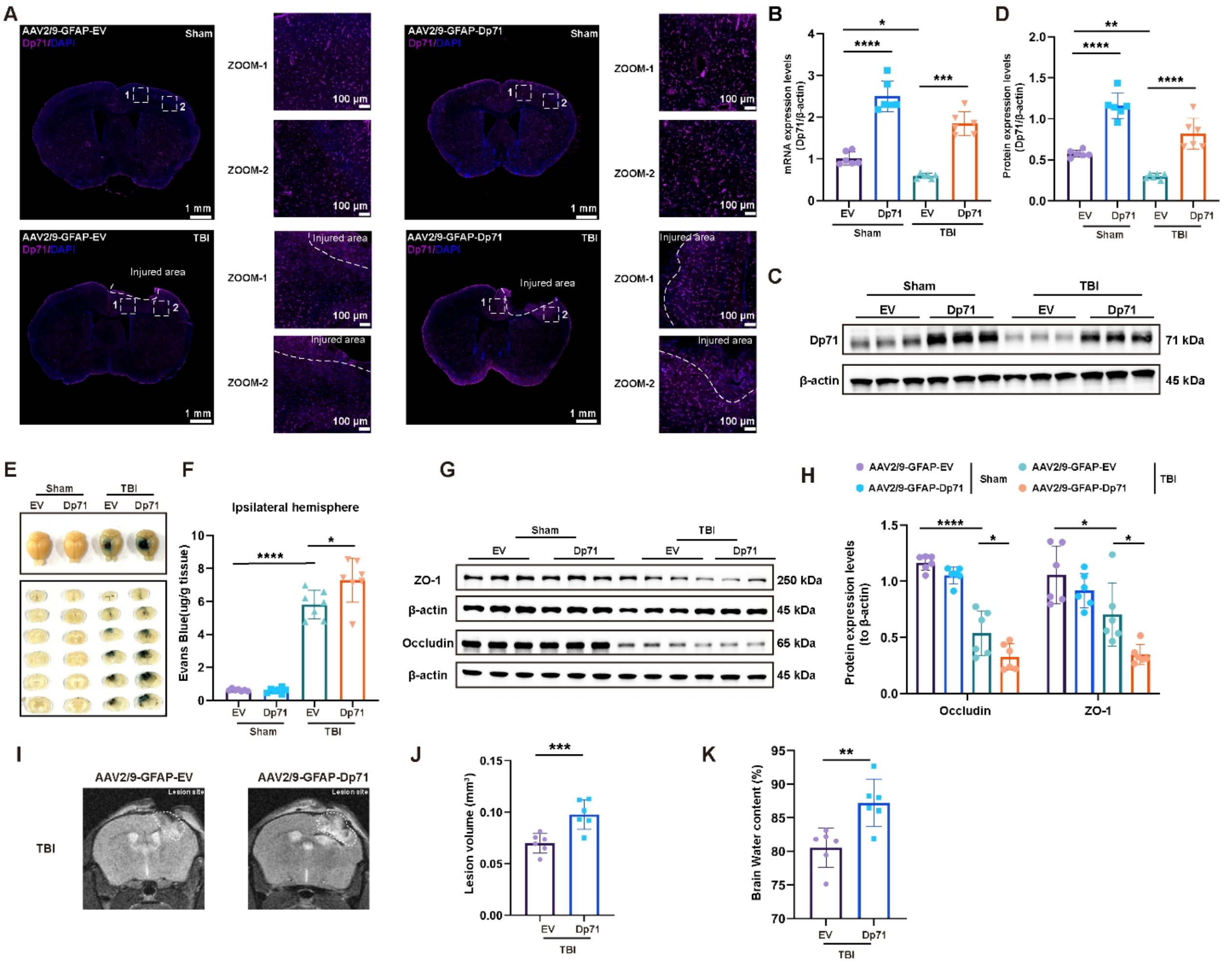

Fig. S4. Astrocyte-specific overexpression of Dp71 exacerbates secondary BBB damage after TBI.

A. Immunofluorescence staining analysis of Dp71 expression in mouse brain (n = 6 per group).
B. Real-time PCR analysis of Dp71 mRNA in the injured area of mouse brain 3 days after TBI (n = 6 per group, two-way ANOVA).
C, D. Western blot analysis of Dp71 protein expression levels in the injured area of mouse brain (n = 6 per group, two-way ANOVA).
E, F. Evans blue staining analysis of BBB permeability in mice with astrocyte-specific Dp71 overexpression at 3 days after TBI (n = 7 per group, two-way ANOVA).
G, H. Western blot analysis of ZO-1 and Occludin (BBB permeability markers) protein expression levels 3 days after TBI (n = 6 per group, two-way ANOVA).
I, J. MRI analysis of brain edema and lesion volume (n = 6 per group, Student's t-test).
K. Assessment of brain edema in mice 3 days after TBI by quantifying brain water content (n = 6 per group, Student's t-test).
Results are expressed as means ± SD. *P < 0.05, **P < 0.01, ***P < 0.001, ****P < 0.0001, NS, not significant.

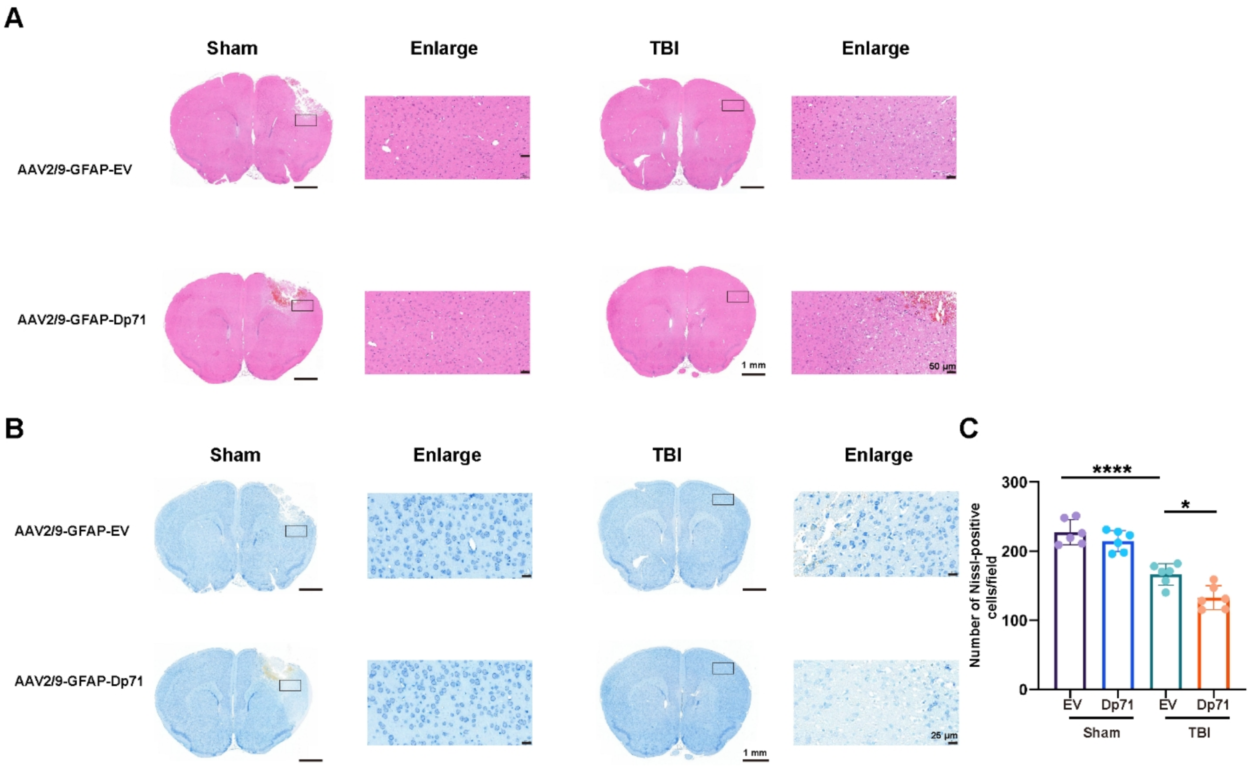

Fig. S5. Astrocyte-specific Dp71 overexpression exacerbates TBI-induced brain injury and neuronal loss in mice.

A. HE staining in the perilesional cortex of Sham and TBI mice. Scale bars, 1 mm for low-magnification images, 50 μm for enlarged images (n = 6 per group).
B, C. Nissl staining in the perilesional cortex. Scale bars, 1 mm for low-magnification images, 25 μm for enlarged images (n = 6 per group, two-way ANOVA).
Results are expressed as means ± SD. *P < 0.05, **P < 0.01, ***P < 0.001, ****P < 0.0001, NS, not significant.

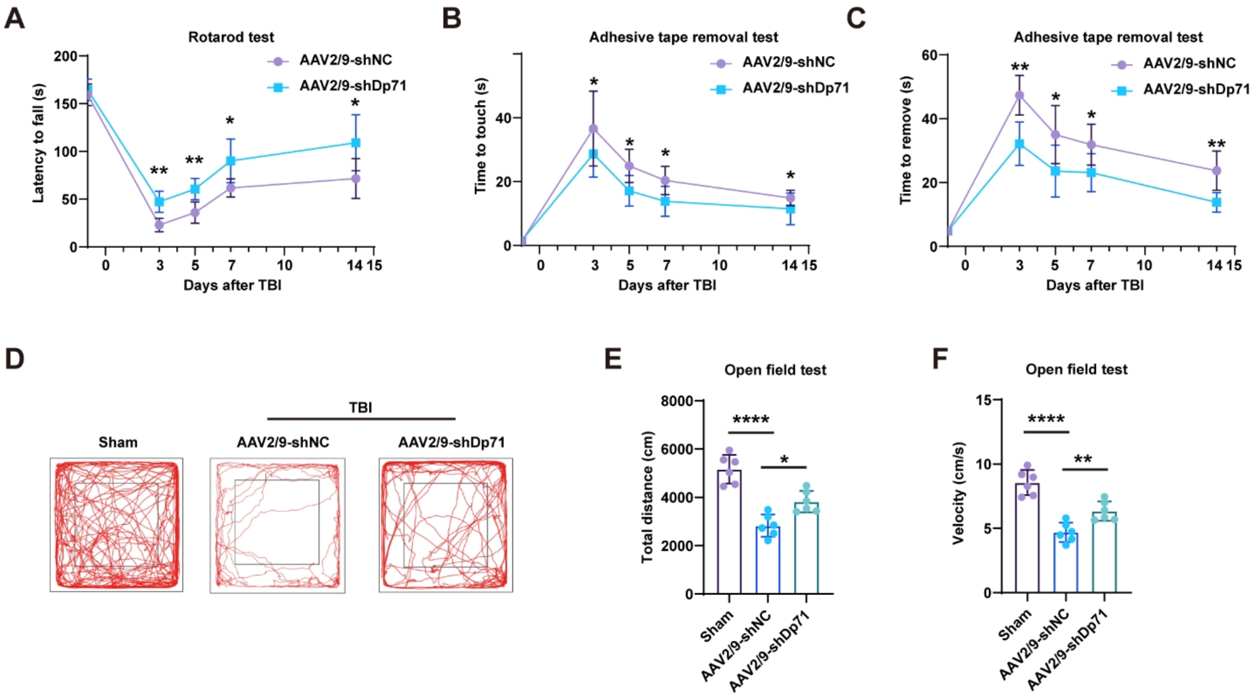

Fig. S6. Knockdown of Dp71 in astrocytes alleviates neurological impairment after TBI.

A. Latency to fall in the rotarod test (n = 6 per group; Student's t-test).

B. Time to touch the tape and **C.** Time to remove the tape in the adhesive tape removal test (n = 6 per group; Student's t-test).

D. Representative trajectory in the open field test.

E, F. Quantitative analysis of total distance and velocity (n = 6 per group; one-way ANOVA). Results are expressed as means ± SD. *P < 0.05, **P < 0.01, ***P < 0.001, ****P < 0.0001, NS, not significant.

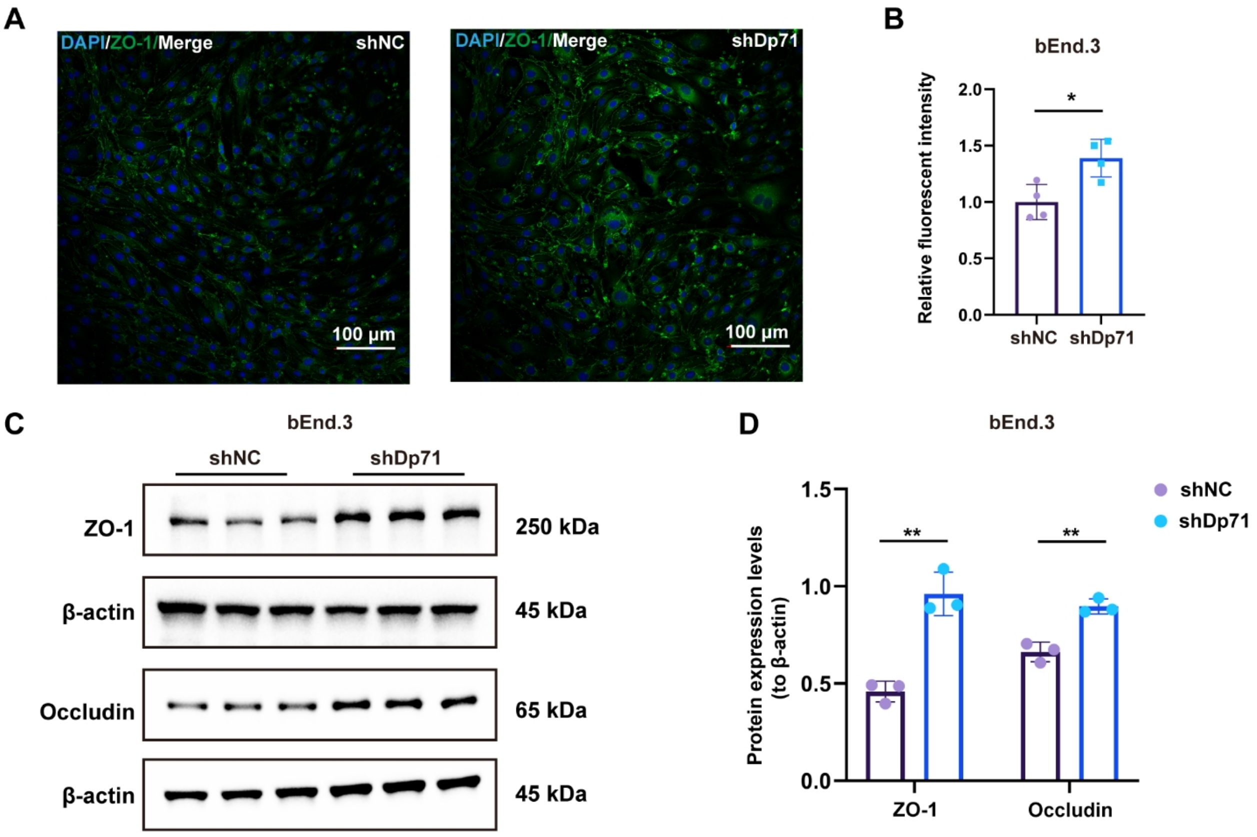

Fig. S7. Dp71-knockdown astrocytes preserve the expression of tight junction proteins ZO-1 and Occludin in bEnd.3 endothelial cells via transwell co-culture.

A, B. Immunofluorescence staining of ZO-1 in bEnd.3 cells co-cultured with shNC or shDp71 astrocytes. Scale bar, 100 μm (n = 4 per group, Student's t-test).

C, D. Western blot analysis of ZO-1 and Occludin in bEnd.3 cells co-cultured with shNC or shDp71 astrocytes (n = 3 per group, Student's t-test).

Results are expressed as means ± SD. *P < 0.05, **P < 0.01, ***P < 0.001, ****P < 0.0001, NS, not significant.

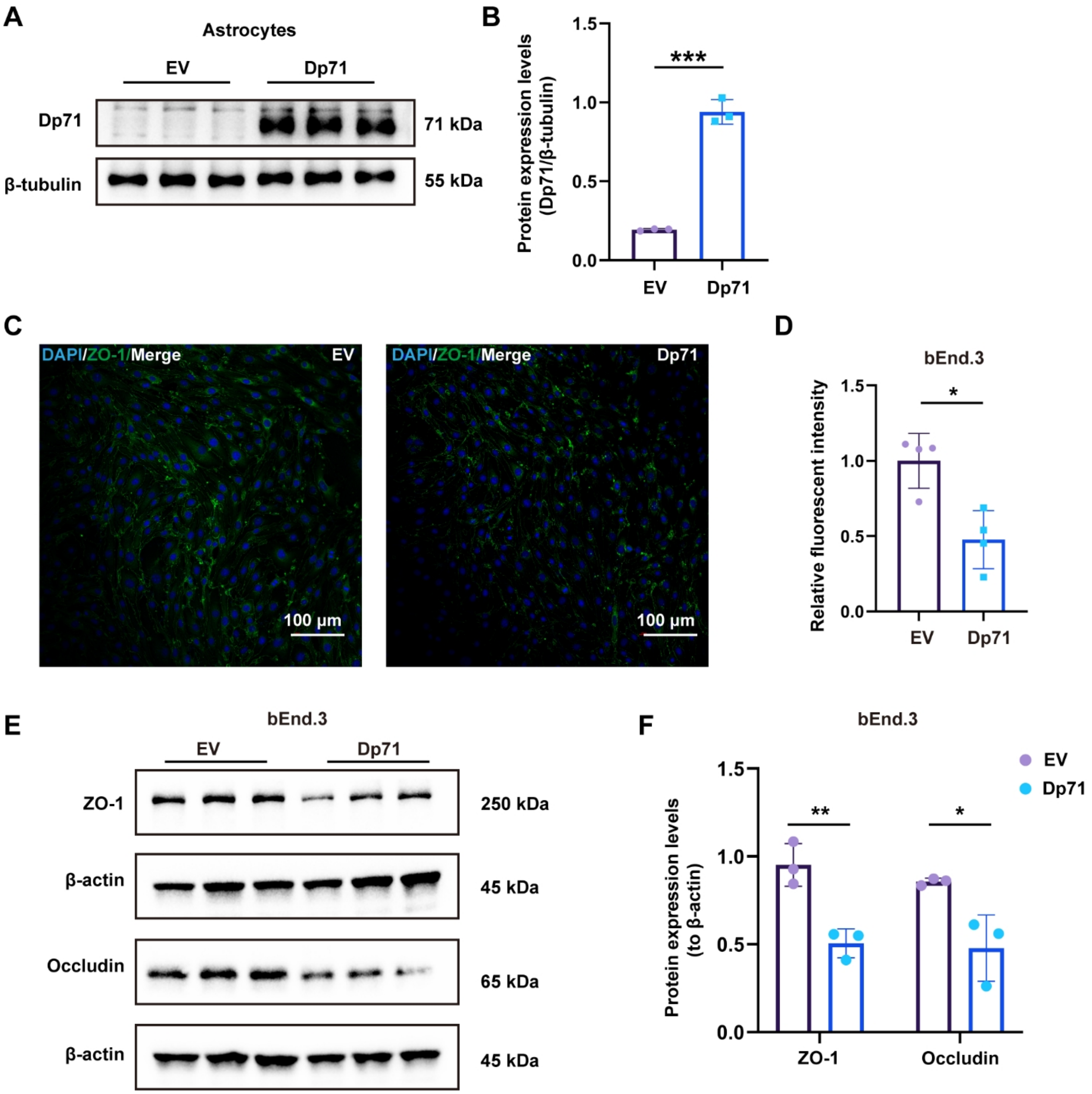

Fig. S8. Dp71-overexpressing astrocytes reduce the expression of tight junction proteins ZO-1 and Occludin in bEnd.3 endothelial cells via transwell co-culture.

A, B. Western blot analysis of Dp71 expression in empty vector (EV) and Dp71-overexpressing (Dp71) astrocytes (n = 3 per group, Student's t-test).
C, D. Immunofluorescence staining of ZO-1 in bEnd.3 cells co-cultured with EV or Dp71 astrocytes. Scale bar, 100 μm (n = 4 per group; Mann-Whitney U test).
E, F. Western blot analysis of ZO-1 and Occludin in bEnd.3 cells co-cultured with EV or Dp71 astrocytes (n = 3 per group, Student's t-test).
Results are expressed as means ± SD. *P < 0.05, **P < 0.01, ***P < 0.001, ****P < 0.0001, NS, not significant.

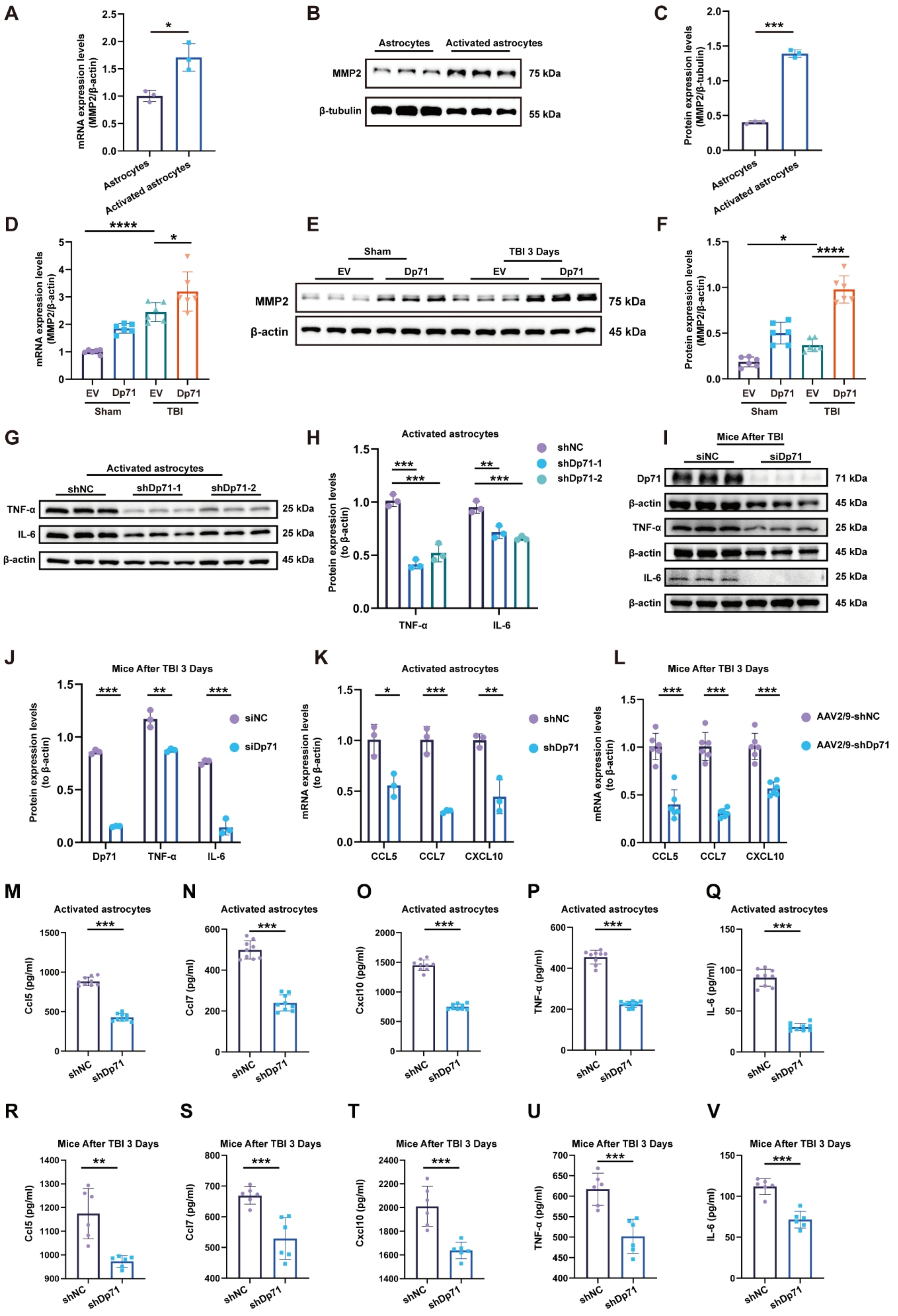
A
B
Astrocytes
Activated astrocytes
MMP2
β-tubulin
75 kDa
55 kDa
C
mRNA expression levels (MMP2/β-actin)
Protein expression levels (MMP2/β-tubulin)
D
E
F
Sham
TBI 3 Days
EV
Dp71
TBI
β-actin
45 kDa
mRNA expression levels (MMP2/β-actin)
Protein expression levels (MMP2/β-actin)
G
H
I
Activated astrocytes
shNC
shDp71-1
shDp71-2
TNF-α
IL-6
25 kDa
Protein expression levels (to β-actin)
Mice After TBI
siNC
siDp71
71 kDa
J
K
L
Mice After TBI 3 Days
CCL5
CCL7
CXCL10
mRNA expression levels (to β-actin)
AAV2/9-shNC
AAV2/9-shDp71
M
N
O
P
Q
Ccl5 (pg/ml)
Ccl7 (pg/ml)
Cxcl10 (pg/ml)
TNF-α (pg/ml)
IL-6 (pg/ml)
R
S
T
U
V

Fig. S9. Knockdown of Dp71 in astrocytes attenuates the release of inflammatory factors.

A. Real-time PCR analysis of MMP2 mRNA expression in astrocytes and activated astrocytes (n = 3 per group, Student's t-test).
B, C. Western blot analysis of MMP2 protein expression in astrocytes and activated astrocytes (n = 3 per group, Student's t-test).
D. Real-time PCR analysis of MMP2 mRNA expression in the injured area of mouse brain 3 days after TBI (n = 6 per group, two-way ANOVA).
E, F. Western blot analysis of MMP2 protein expression levels in the injured area of mouse brain (n = 6 per group, two-way ANOVA).
G, H. Western blot analysis of inflammatory protein expression in activated astrocytes (n = 3 per group, one-way ANOVA).
I, J. Western blot analysis of inflammatory protein expression in TBI-injured brain regions of mice injected with cholesterol-conjugated siNC and siDp71 (n = 3 per group, Student's t-test).
K. Real-time PCR analysis of CCL5, CCL7, and CXCL10 mRNA expression in activated astrocytes (n = 3 per group, one-way ANOVA).
L. Real-time PCR analysis of CCL5, CCL7, and CXCL10 mRNA expression in shNC and shDp71 mice after TBI (n = 3 per group, Student's t-test).
M-Q. ELISA analysis of CCL5, CCL7, CXCL10, TNF-α, and IL-6 protein concentrations in shNC and shDp71 astrocytes (n = 9 per group, Student's t-test).
R-V. ELISA analysis of CCL5, CCL7, CXCL10, TNF-α, and IL-6 protein concentrations in shNC and shDp71 mice 3 days after TBI (n = 6 per group, Student's t-test).
Results are expressed as means ± SD. *P < 0.05, **P < 0.01, ***P < 0.001, ****P < 0.0001, NS, not significant.

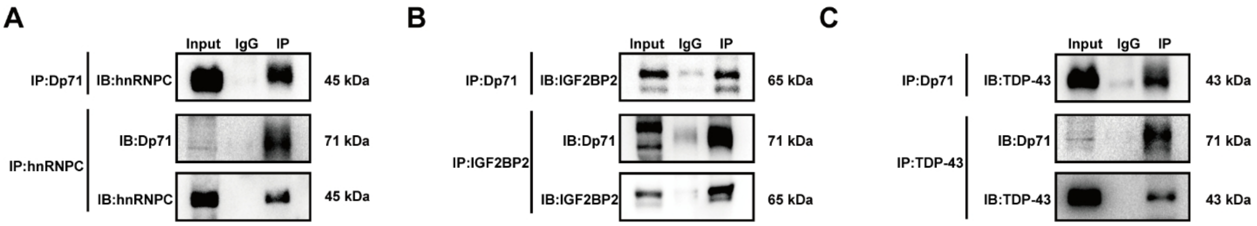

Fig. S10. Dp71 interacts with m^{6}A-related proteins.

A. IB analysis of co-IP for Dp71 and hnRNPC in astrocytes (n = 3).
B. IB analysis of co-IP for Dp71 and IGF2BP2 in astrocytes (n = 3).
C. IB analysis of co-IP for Dp71 and TDP-43 in astrocytes (n = 3).

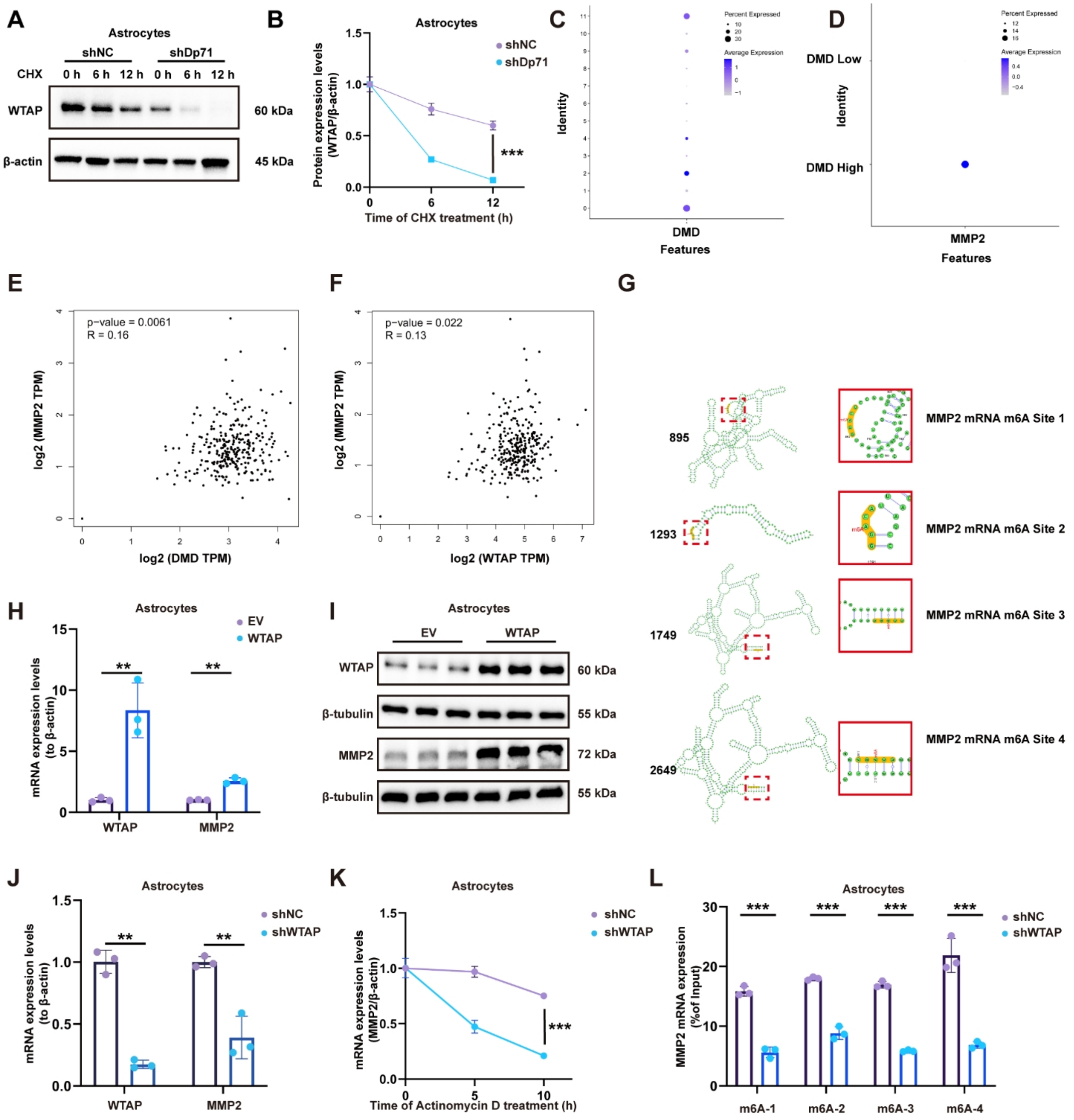

Fig. S11. WTAP regulates MMP2 expression via m6A modification of its mRNA in astrocytes.

A, B. Western blot analysis of WTAP protein expression in shNC and shDp71 astrocytes treated with CHX for 0, 6, and 12 hours (n = 3 per group, Student's t-test).
C. scRNA-seq analysis of DMD expression in astrocytes, stratified into DMD Low and DMD High subgroups.
D. scRNA-seq analysis of MMP2 expression and its correlation with DMD expression in astrocytes.
E, F. Correlation analysis of DMD, WTAP, and MMP2 mRNA expression in the cerebral cortex.
G. Visualization of m^{6}A methylation sites on MMP2 mRNA.

H. Real-time PCR analysis of WTAP and MMP2 mRNA expression in EV and WTAP-overexpression astrocytes (n = 3 per group, Student's t-test).
I. Western blot analysis of WTAP and MMP2 protein expression in EV and WTAP-overexpression astrocytes (n = 3 per group, Student's t-test).
J. Real-time PCR analysis of WTAP and MMP2 mRNA expression in shNC and shWTAP astrocytes (n = 3 per group, Student's t-test).
K. Real-time PCR analysis of MMP2 mRNA stability in shNC and shWTAP astrocytes. Astrocytes were treated with Actinomycin D, and RNA was collected at different time points (n = 3 per group, Student's t-test).
L. MeRIP analysis of m^6A modification levels on MMP2 mRNA in shNC and shWTAP astrocytes (n = 3 per group, Student's t-test).
Results are expressed as means ± SD. *P < 0.05, **P < 0.01, ***P < 0.001, ****P < 0.0001, NS, not significant.

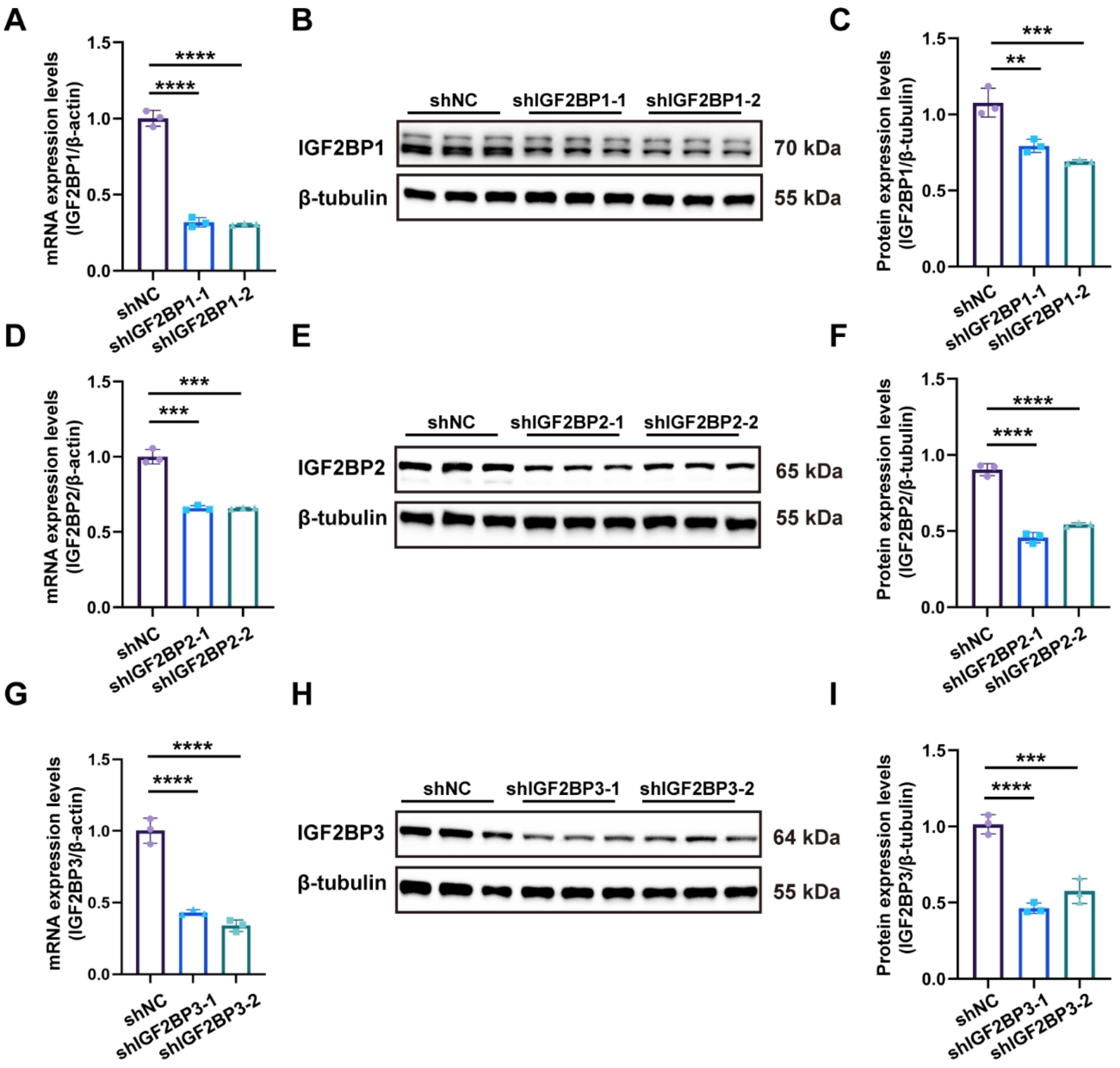

Fig. S12. IGF2BP1/2/3 knockdown via lentivirus robustly suppresses their mRNA and protein expression in astrocytes.

A. Real-time PCR analysis of IGF2BP1 mRNA expression levels in astrocytes transduced with lentiviral shNC, shIGF2BP1-1, and shIGF2BP1-2 (n = 3 per group, one-way ANOVA).
B, C. Western blot analysis of IGF2BP1 protein expression in astrocytes transduced with lentiviral shNC, shIGF2BP1-1, and shIGF2BP1-2 (n = 3 per group, one-way ANOVA).
D. Real-time PCR analysis of IGF2BP2 mRNA expression levels in astrocytes transduced with lentiviral shNC, shIGF2BP2-1, and shIGF2BP2-2 (n = 3 per group, one-way ANOVA).
E, F. Western blot analysis of IGF2BP2 protein expression in astrocytes transduced with lentiviral shNC, shIGF2BP2-1, and shIGF2BP2-2 (n = 3 per group, one-way ANOVA).
G. Real-time PCR analysis of IGF2BP3 mRNA expression levels in astrocytes transduced with lentiviral shNC, shIGF2BP3-1, and shIGF2BP3-2 (n = 3 per group, one-way ANOVA).
H, I. Western blot analysis of IGF2BP3 protein expression in astrocytes transduced with lentiviral shNC, shIGF2BP3-1, and shIGF2BP3-2 (n = 3 per group, one-way ANOVA).
Results are expressed as means ± SD. *P < 0.05, **P < 0.01, ***P < 0.001, ****P < 0.0001, NS, not significant.

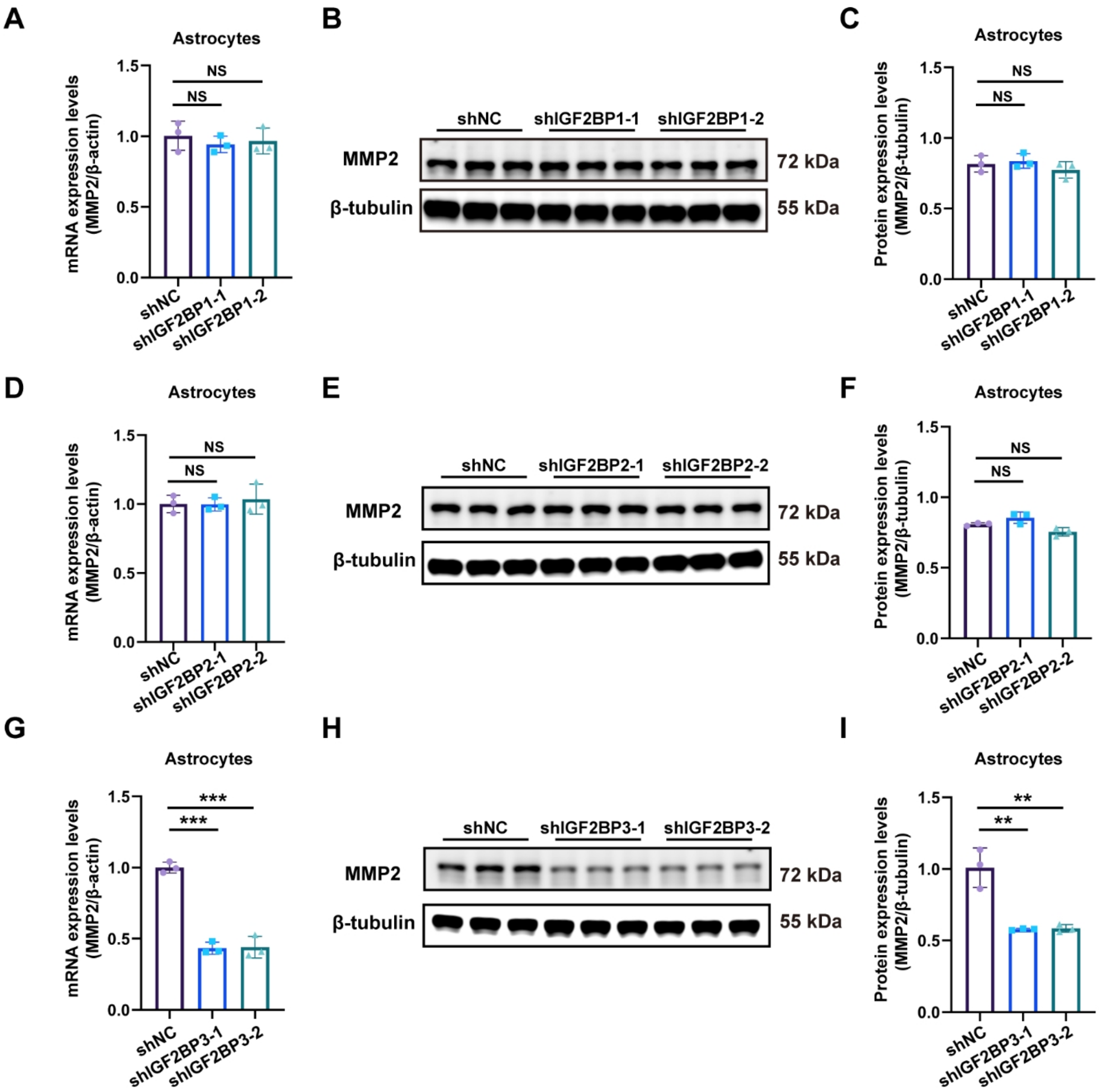

Fig. S13. Knockdown of IGF2BP3 (but not IGF2BP1 or IGF2BP2) reduces MMP2 expression in astrocytes.

A. Real-time PCR analysis of MMP2 mRNA expression levels in shNC, shIGF2BP1-1, and shIGF2BP1-2 astrocytes (n = 3 per group, one-way ANOVA).
B, C. Western blot analysis of MMP2 protein expression in shNC, shIGF2BP1-1, and shIGF2BP1-2 astrocytes (n = 3 per group, one-way ANOVA).
D. Real-time PCR analysis of MMP2 mRNA expression levels in shNC, shIGF2BP2-1, and shIGF2BP2-2 astrocytes (n = 3 per group, one-way ANOVA).
E, F Western blot analysis of MMP2 protein expression in shNC, shIGF2BP2-1, and shIGF2BP2-2 astrocytes (n = 3 per group, one-way ANOVA).
G. Real-time PCR analysis of MMP2 mRNA expression levels in shNC, shIGF2BP3-1, and shIGF2BP3-2 astrocytes (n = 3 per group, one-way ANOVA).

H, I. Western blot analysis of MMP2 protein expression in shNC, shIGF2BP3-1, and shIGF2BP3-2 astrocytes (n = 3 per group, one-way ANOVA).
Results are expressed as means ± SD. *$P < 0.05$, **$P < 0.01$, ***$P < 0.001$, ****$P < 0.0001$, NS, not significant.

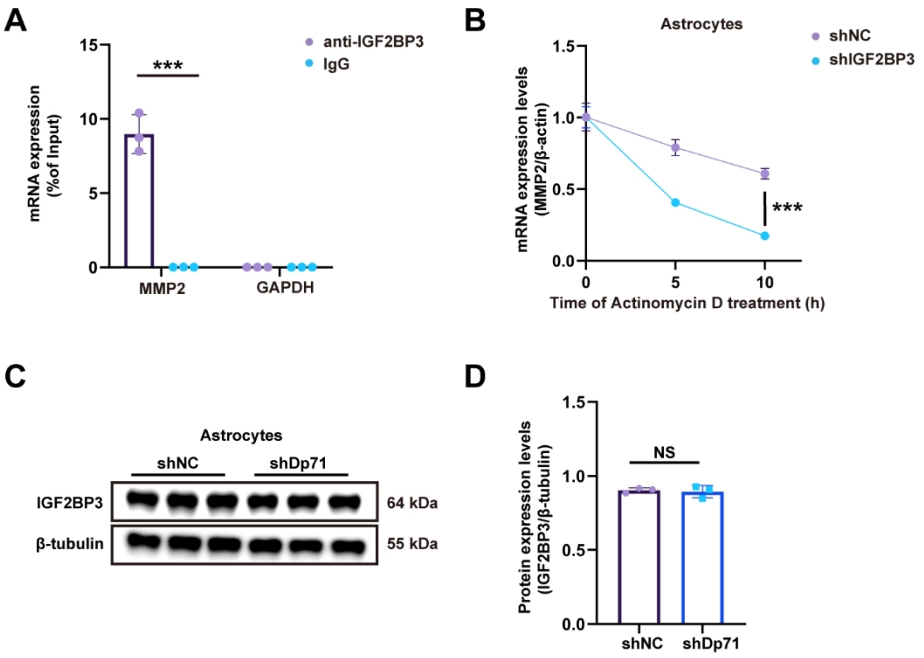

Fig. S14. IGF2BP3 binds and stabilizes MMP2 mRNA in astrocytes.

A. RIP analysis of the interaction between IGF2BP3 and MMP2 mRNA in astrocytes (n = 3 per group, Student's t-test).

B. mRNA stability analysis of MMP2 in shNC and shIGF2BP3 astrocytes treated with Actinomycin D for 0, 5, and 10 hours (n = 3 per group, Student's t-test).

C, D. Western blot analysis of IGF2BP3 protein expression in shNC and shDp71 astrocytes (n = 3 per group, Student's t-test).

Results are expressed as means ± SD. *P < 0.05, **P < 0.01, ***P < 0.001, ****P < 0.0001, NS, not significant.

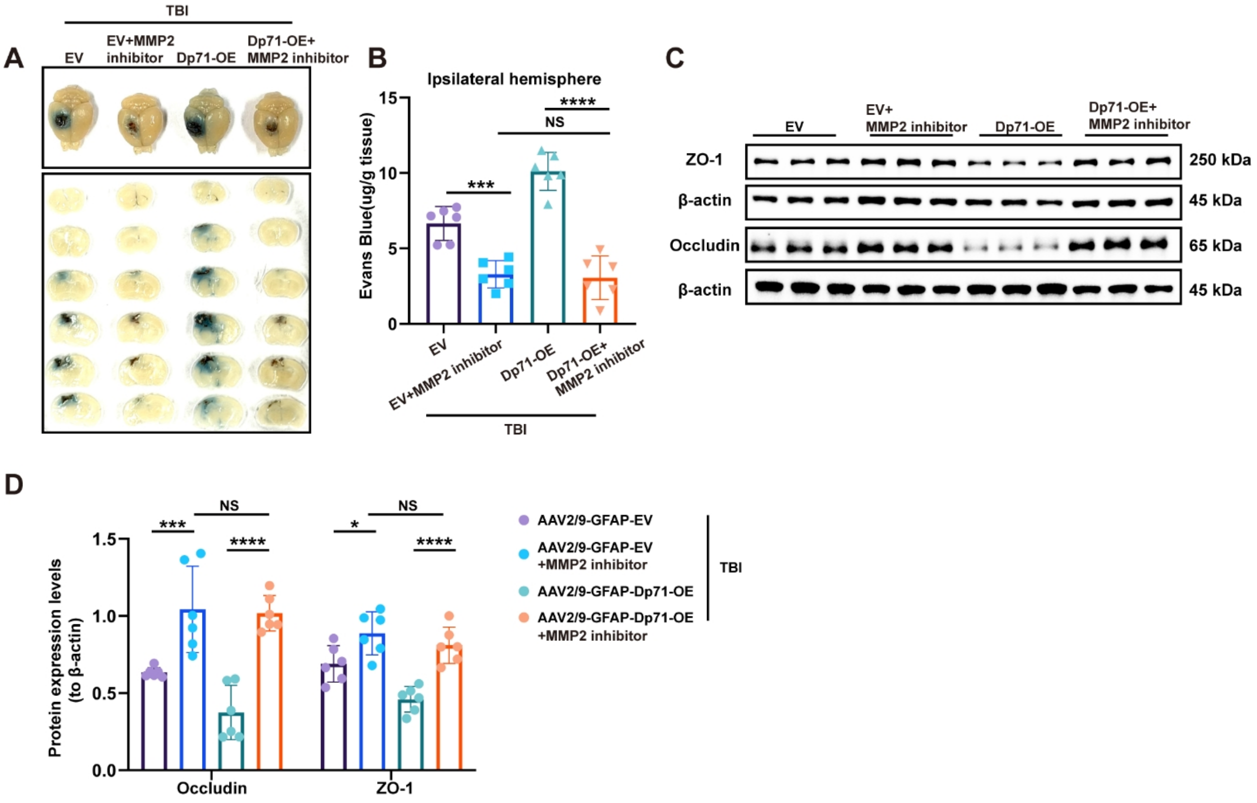

Fig. S15. MMP2 inhibition attenuates astrocyte-specific Dp71 overexpression-exacerbated brain damage following traumatic brain injury.

A, B. Evans blue staining analysis of BBB permeability in mice after TBI (n = 6 per group, two-way ANOVA).

C, D. Western blot analysis of Occludin and ZO-1 protein expression in mice after TBI (n = 6 per group, two-way ANOVA).

Results are expressed as means ± SD. *P < 0.05, **P < 0.01, ***P < 0.001, ****P < 0.0001, NS, not significant.

A

	Hydrodynamic size (nm)	PDI
TRAM	99.07±3.54	0.237±0.008
TRAM@siNC	104.05±9.11	0.19±0.028
TRAM@siDp71	103.47±0.56	0.228±0.009

B

	Loading efficiency（%）		Encapsulation efficiency（%）	
	Cy5.5	siRNA	Cy5.5	siRNA
TRAM@siNC	1.13	1.36	90.4	90.67
TRAM@siDp71	1.16	1.27	92.8	85

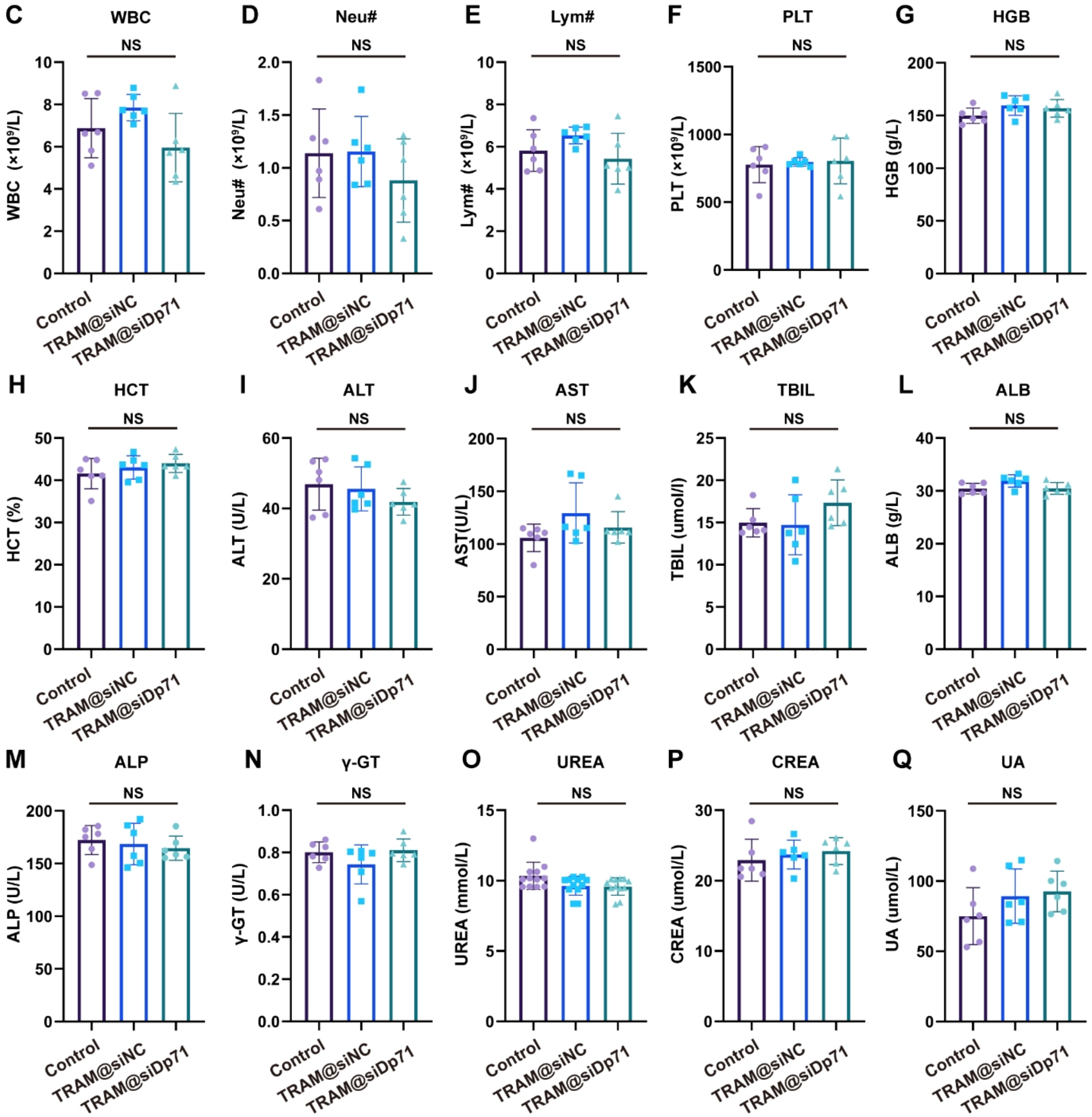

Fig. S16. Physicochemical characterization and in vivo safety evaluation of TRAM@siDp71 nanoparticles.

A. Hydrodynamic size and polydispersity index (PDI) of TRAM@siNC and TRAM@siDp71 nanovesicles.

B. Left panel: Loading efficiency (LE%) of siDp71 and Cy5.5 in TRAM@siNC and TRAM@siDp71 nanovesicles. Right panel: Encapsulation efficiency (EE%) of siDp71 and Cy5.5 in TRAM@siNC and TRAM@siDp71 nanovesicles.
C-Q. Hematological and biochemical analyses in mice treated with Control, TRAM@siNC, or TRAM@siDp71, including white blood cells (WBC), neutrophils (Neu#), lymphocytes (Lym#), platelets (PLT), hemoglobin (HGB), hematocrit (HCT), and liver/renal function markers (ALT, AST, TBIL, ALB, ALP, γ-GT, UREA, CREA, UA) ($n = 6$ per group). For normally distributed indicators (WBC, Neu#, Lym#, PLT, HGB, HCT, ALT, ALB, ALP, CREA, UA): one-way ANOVA with Tukey's multiple comparisons test was used. For non-normally distributed indicators (AST, TBIL, γ-GT, UREA): Kruskal-Wallis H test with Dunn's multiple comparisons test was applied.
Results are expressed as means ± SD. *$P < 0.05$, **$P < 0.01$, ***$P < 0.001$, ****$P < 0.0001$, NS, not significant.

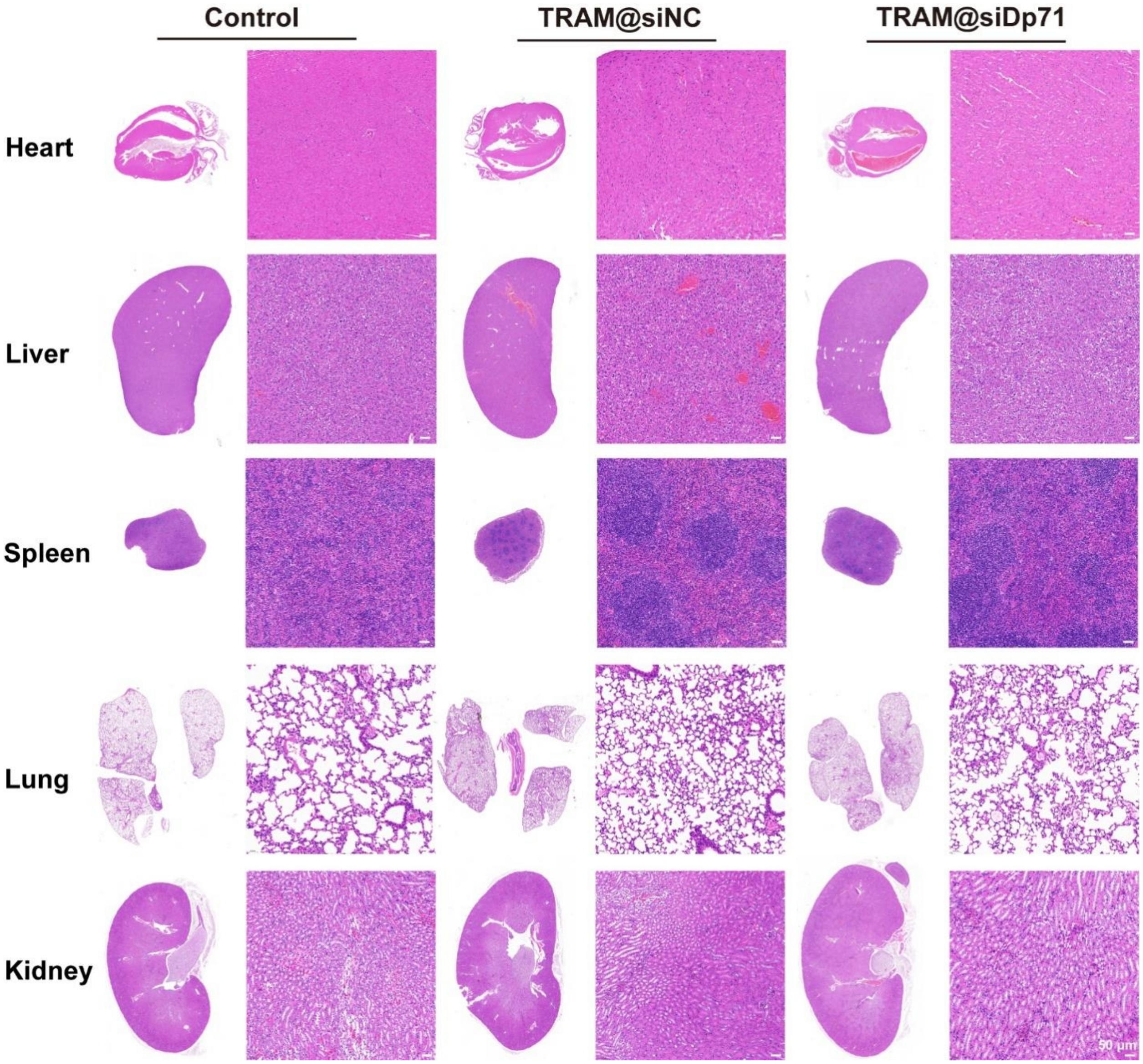

Fig. S17. The biocompatibility results of different organs.

Histological sections stained with H&E from different organs after nanovesicle treatment, scale bars, 50 μm.

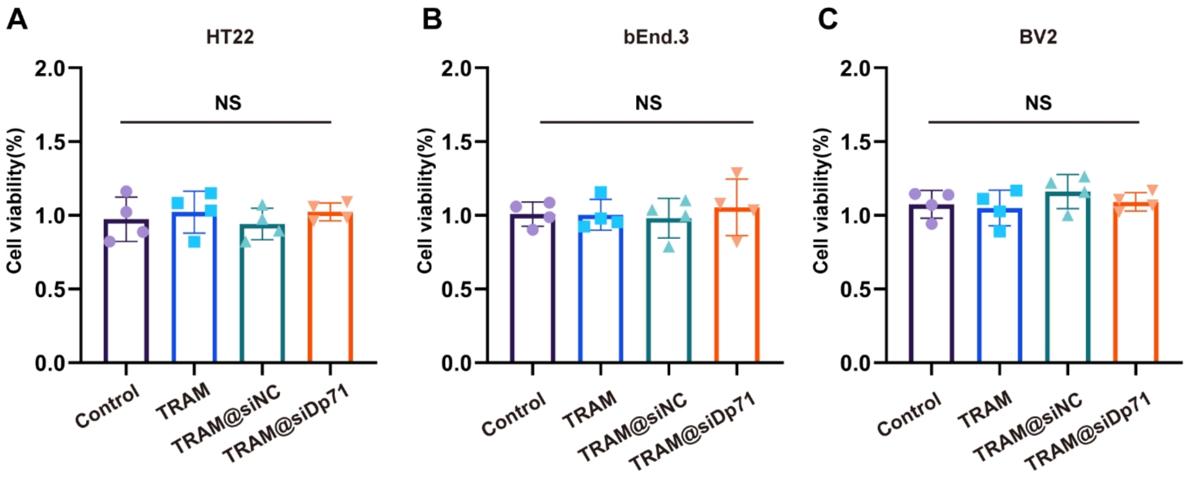

Fig. S18. TRAM@siDp71 nanoparticles exhibit no cytotoxicity in HT22, bEnd.3, and BV2 cells.

A-C. Cell viability analysis of HT22 (neurons), bEnd.3 (brain endothelial cells), and BV2 (microglia) treated with Control, TRAM, TRAM@siNC, or TRAM@siDp71 ($n = 4$ per group; one-way ANOVA).

Results are expressed as means ± SD. *$P < 0.05$, **$P < 0.01$, ***$P < 0.001$, ****$P < 0.0001$, NS, not significant.

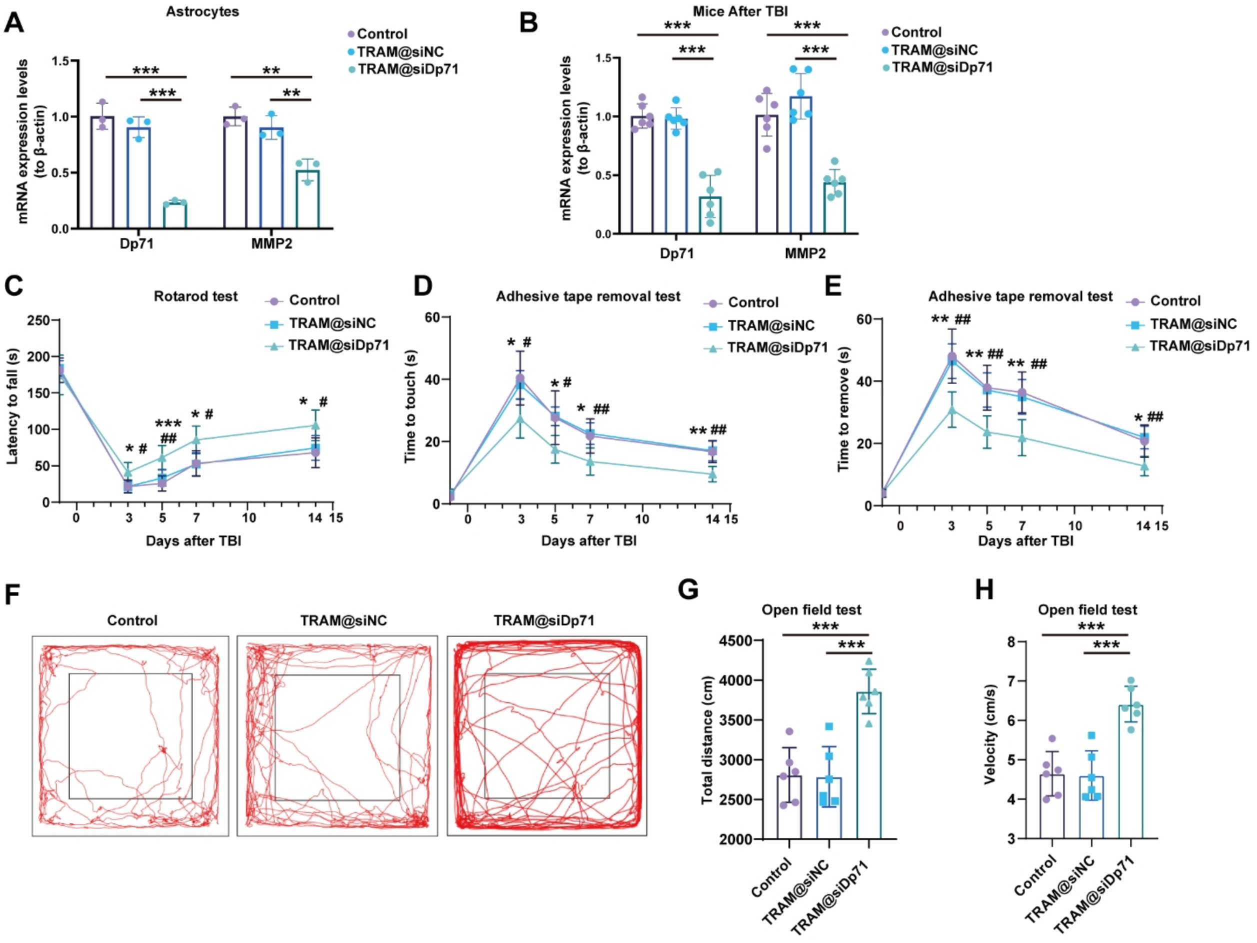

Fig. S19 TRAM@siDp71 is associated with improved neurological functional recovery in mice after TBI.

A. Real-time PCR analysis of Dp71 and MMP2 mRNA expression levels in astrocytes treated with TRAM@siNC or TRAM@siDp71 (n = 3 per group, one-way ANOVA).
B. Real-time PCR analysis of Dp71 and MMP2 mRNA expression levels in TBI mice intravenously injected with TRAM@siNC or TRAM@siDp71 (n = 6 per group, one-way ANOVA).
C. Latency to fall in the rotarod test (n = 6 per group, one-way ANOVA).
D. Adhesive tape removal test (time to touch) for somatosensory function (n = 6 per group, one-way ANOVA.).
E. Adhesive tape removal test (time to remove) for somatosensory function (n = 6 per group, one-way ANOVA).
F. Representative trajectory in the open field test.
G, H. Quantitative analysis of total distance and velocity (n = 6 per group; one-way ANOVA). Results are expressed as means ± SD. *P < 0.05, **P < 0.01, ***P < 0.001, ****P <0.0001 versus TRAM@siDp71+TRAM@siNC; $^{\#}$P < 0.05, $^{\#\#}$P < 0.01, $^{\#\#\#}$P < 0.001, $^{\#\#\#\#}$P <0.0001, versus TRAM@siDp71+Control. NS, not significant.
